# Multifunctional dietary approach reduces intestinal inflammation in relation with changes in gut microbiota composition in subjects at cardiometabolic risk: the SINFONI project

**DOI:** 10.1080/19490976.2024.2438823

**Published:** 2024-12-22

**Authors:** Hugo Hornero-Ramirez, Arianne Morissette, Bruno Marcotte, Armelle Penhoat, Béryle Lecomte, Baptiste Panthu, Jacob Lessard Lord, Florence Thirion, Laurie Van-Den-Berghe, Emilie Blond, Chantal Simon, Cyrielle Caussy, Nathalie Feugier, Joël Doré, Philippe Sanoner, Alexandra Meynier, Yves Desjardins, Geneviève Pilon, André Marette, Patrice D. Cani, Martine Laville, Sophie Vinoy, Marie-Caroline Michalski, Julie-Anne Nazare

**Affiliations:** a Centre de Recherche en Nutrition Humaine - Rhône-Alpes, INSERM, INRAe, Université Claude Bernard Lyon1, Hospices Civils de Lyon, Pierre Bénite, France; b Laboratoire CarMeN, INSERM U.1060, INRAe U. 1397, Université Claude Bernard Lyon1, Pierre Bénite, France; c Institute of Nutrition and Functional Foods (INAF), Faculty of Agriculture and Food Sciences, Laval University, Québec, QC, Canada; d Nutrition, Health and Society Centre (NUTRISS), INAF, Laval University, Québec, QC, Canada; e Department of Plant Science, Faculty of Agriculture and Food Sciences, Laval University, Québec, QC, Canada; f INRAE, MGP, Université Paris-Saclay, Jouy-en-Josas, France; g Biochemistry Department, Hospices Civils de Lyon, Pierre-Bénite, France; h Endocrinology, Diabetes and Nutrition Department, Lyon South Hospital, Civil Hospices of Lyon, Pierre-Bénite, France; i iSymrise-Diana Food SAS, R&D, Naturals Food & Beverage, Rennes, France; j Nutrition Research, Paris-Saclay Tech Center, Mondelez International R&D, Saclay, France; k Department of Medicine, Faculty of Medicine, Québec Heart and Lung Institute, Université Laval, Québec, Canada; l Centre Nutrition, santé et société (NUTRISS), Institute of Nutrition and Functional Foods (INAF), Université Laval, Québec, Canada; m UCLouvain, Université catholique de Louvain, Louvain Drug Research Institute, (LDRI) Metabolism and Nutrition Research Group (MNUT), Brussels, Belgium; n Louvain Drug Research Institute; Walloon Excellence in Life Sciences and BIOtechnology (WELBIO), WELBIO department, WEL Research Institute, Wavre, Belgium; o UCLouvain, Université catholique de Louvain, Institute of Experimental and Clinical Research (IREC), Brussels, Belgium

**Keywords:** Polyphenols, omega 3, endotoxemia, cardiometabolic risk, branched chain amino-acids, intestinal inflammation

## Abstract

The development of cardiometabolic (CM) diseases is associated with chronic low-grade inflammation, partly linked to alterations of the gut microbiota (GM) and reduced intestinal integrity. The SINFONI project investigates a multifunctional (MF) nutritional strategy’s impact combining different bioactive compounds on inflammation, GM modulation and CM profile. In this randomized crossover-controlled study, 30 subjects at CM-risk consumed MF cereal-products, enriched with polyphenols, fibers, slowly-digestible starch, omega-3 fatty acids or Control cereal-products (without bioactive compounds) for 2 months. Metabolic endotoxemia (lipopolysaccharide (LPS), lipopolysaccharide-binding protein over soluble cluster of differentiation-14 (LBP/sCD14), systemic inflammation and cardiovascular risk markers, intestinal inflammation, CM profile and response to a one-week fructose supplementation, were assessed at fasting and post mixed-meal. GM composition and metabolomic analysis were conducted. Mixed linear models were employed, integrating time (pre/post), treatment (MF/control), and sequence/period. Compared to control, MF intervention reduced intestinal inflammation (fecal calprotectin, *p* = 0.007) and endotoxemia (fasting LPS, *p* < 0.05), without alteration of systemic inflammation. MF decreased serum branched-chain amino acids compared to control (*p* < 0.05) and increased *B.ovatus*, *B.uniformis*, *A.butyriciproducens* and unclassified *Christensenellaceae.CAG-74* (*p* < 0.05). CM markers were unchanged. A 2-month dietary intervention combining multiple bioactive compounds improved intestinal inflammation and induced GM modulation. Such strategy appears as an effective strategy to target low-grade inflammation through multi-target approach.

## Introduction

Cardiometabolic (CM) diseases, including obesity, type 2 diabetes (T2D), and cardiovascular diseases have become a global public health challenge, leading to escalating healthcare costs primarily focused on treatment rather than prevention. A state of low-grade chronic inflammation, characterized by persistent inflammation, serves as a common underlying factor in various chronic inflammatory diseases.^1^ As reported by Van Den Brink et al, inflammation is a sequential, multi-organ, systemic process resulting from various induced metabolic stresses that can be addressed by a wide range of distinct biomarkers.^2^ The role of gut microbiota in obesity susceptibility and in the development of metabolic complications such as adiposity and insulin resistance has also gathered increasing attention, notably in relation to systemic inflammation.^3^ Recent evidence links part of this inflammatory state to disturbances in gut microbiota composition, function, and related compromised gut barrier.^4–6^ An alteration of the intestinal barrier could result in the translocation into the bloodstream of gut microbiota-derived lipopolysaccharide (LPS), also known as metabolic endotoxemia.^7^ This phenomenon has been described as a crucial factor in both initiating and advancing inflammation and metabolic diseases.^7^ Moreover, the interplay between dietary compounds and the gut has been identified as a pivotal component with both causative and therapeutic potential in managing low-grade inflammation and metabolic abnormalities.^8,9^ Some dietary components, such as plant-derived bioactive compounds, fats or carbohydrates, isolated or combined, and certain dietary patterns beneficially associated with lower inflammation tone and improved insulin sensitivity and CM profile.^8–10^ Nevertheless, discrepancies remain in the literature between epidemiological studies and nutritional interventions regarding the impact of dietary patterns on the inflammatory profile, partly due to the heterogeneity of studied inflammatory markers. Previous works have mainly focused on assessing a single or a limited number of systemic inflammatory markers, while the overall impact of nutritional interventions is multifactorial and impacting complex multi-organ interactions, urging for integrative multiple inflammatory markers analysis.^2,10^ Recent research has also revealed that beyond fasting status, meal and postprandial periods are key features to address the diet-related physiological processes of inflammatory status.^11,12^ However metabolic challenges are still barely used although they allow capture the dynamic metabolic impact in response to dietary interventions.^13^

Previous work from our teams and others has revealed key food bioactive ingredients with high potential to beneficially alter inflammatory status by reducing the production of pro-inflammatory mediators/damaging antioxidants or promoting gut integrity and modulating GM and anti-inflammatory processes in animal and human studies.^14–18^ Polyphenols have demonstrated protective effects against obesity-linked metabolic diseases, particularly insulin resistance and inflammation, potentially by increasing the abundance of specific beneficial gut bacteria such as *Akkermansia muciniphila*.^19,20^ This bacterium has been shown to alleviate intestinal inflammation and improve metabolic health.^19–23^ Similarly, omega-3 fatty acids have been found to reduce low-grade inflammation and improve cardiometabolic health partly by targeting intestinal microbiota.^24^ Post-meal glycemic fluctuations are also a critical determinant of both postprandial and chronic low-grade inflammation, acting partly through oxidative stress.^13^ Altering carbohydrate quality to lower the glycemic index of foods or to slowing starch digestibility is promising in modulating inflammatory profiles, thereby reducing postprandial glycemic excursions in both healthy individuals and those with obesity or type 2 diabetes.^16,25,26^ Additionally, dietary fibers, which are crucial for stabilizing and enhancing gut microbiota function, have a direct effect in the reduction of glycemic excursions and have been associated with decreased endotoxemia, and mitigated cardiovascular disease risk and pro-inflammatory markers.^27–29^

In the SINFONI project, our hypothesis posits that combining selected bioactive compounds potentially targeting simultaneously multiple inflammation-related features such as metabolic endotoxemia, gut integrity, gut microbiota composition as well as postprandial metabolic responses, would be an efficient nutritional strategy for reducing low-grade inflammation and mitigating CM risk factors. In this sense, we previously developed and validated a MF cereal-based product enriched with polyphenols, dietary fibers, slowly digestible starch (SDS) and polyunsaturated fatty acids (PUFAs).^30^ The objective of the SINFONI study is to evaluate the impact of an 8-week MF dietary intervention in at CM-risk individuals on intestinal and low-grade inflammation by a multi-biomarker approach in relation to gut microbiota modulation and CM profile.

## Results

### Participant characteristics

**Figure 1.**
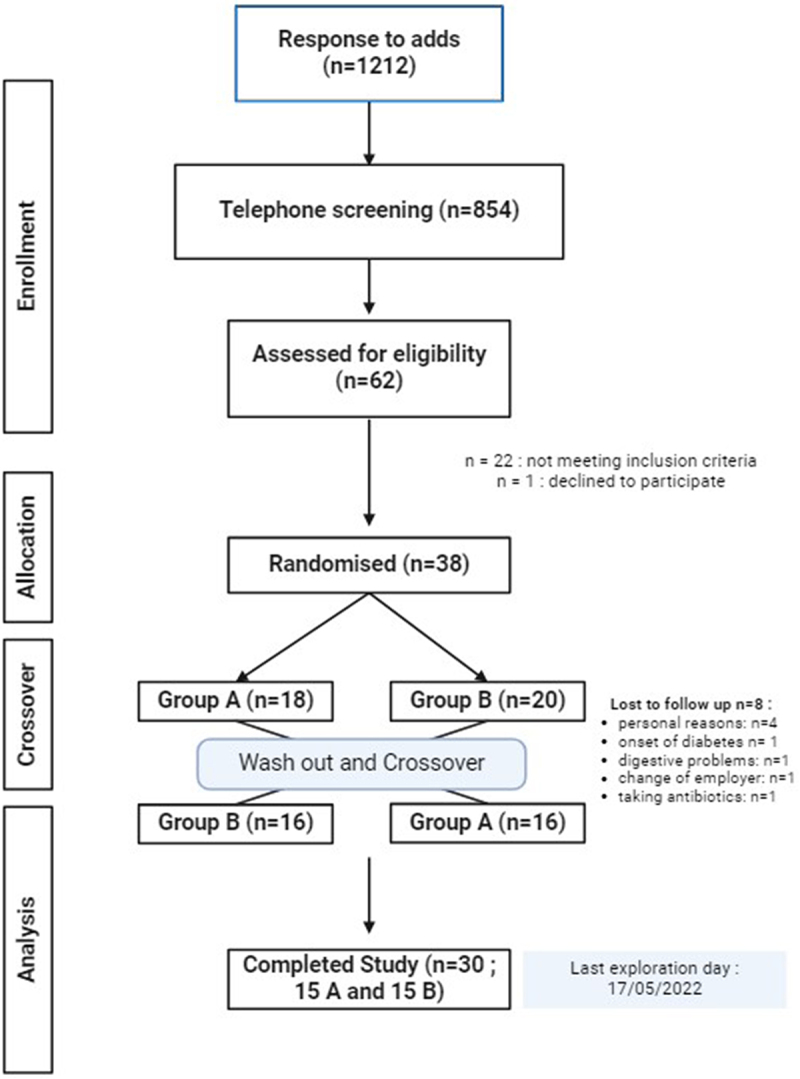
**Figure 1:** SINFONI Consort Flow Diagram

From 1212 individuals who responded to ads and initially screened, 30 CM-risk individuals were included, randomized and completed the study, with a sex ratio of 50% as expected (Figure 1). Of these, 33% of subjects had dyslipidemia (high triglycerides and/or low high-density lipoprotein cholesterol and/or high low-density lipoprotein cholesterol and/or high total cholesterol). Participants did not present fasting hyperglycemia, diabetes, elevated C-Reactive Proteins (CRP), or hypertension (Table 1).

**Table 1.** Baseline characteristics of subjects at inclusion.

	Female			Male		
	*n* = 15			*n* = 15		
Age (years)	43.7	±	8.1	44.4	±	8.5
Weight (kg)	77.7	±	8.8	90.2	±	7.1
Height (m)	1.6	±	0.1	1.7	±	0.1
BMI (kg/m^2^	29.1	±	2.5	28.5	±	1.8
Waist circumference (cm)	97.7	±	5.8	102.4	±	6.1
Hip circumference (cm)	109.8	±	6.2	106.4	±	6.6
Systolic Pressure (mmHg)	124.1	±	13.3	124.8	±	7.8
Diastolic Pressure (mmHg)	74.1	±	8.8	76.3	±	6.9
Glucose (mmol/L)	5.0	±	0.3	4.9	±	0.2
TG (mmol/L)	1.0	±	0.3	1.40	±	0.5
TC (mmol/L)	5.0	±	1.1	5.7	±	1.1
HDL-C (mM)	1.4	±	0.3	1.2	±	0.1
LDL-C (mM)	3.3	±	0.5	3.9	±	0.9
CRP (mg/L)	2.6	±	2.8	3.37	±	4.4
ASAT (UI/L)	21.5	±	4.4	26.9	±	6.4
ALAT (UI/L)	23.6	±	7.2	37.4	±	15.5
GGT (UI/L)	21.7	±	9.6	34.5	±	16.1

Data are expressed as mean ± SD. BMI : body mass index; TG : triglyceride; TC : total cholesterol, HDL-C : high-density lipoprotein cholesterol, LDL-C: low-density lipoprotein cholesterol; CRP : C reactive protein; ASAT : Aspartate transaminase; ALAT : alanine aminotransferase; GGT : gamma-glutamyl transferase. All data were quantified at fasting state.

### Compliance and dietary intake

Compliance with cereal product consumption was 96%. All subjects who completed the study had no variation in caloric or carbohydrate intake, nor in fatty acid and protein or fiber between the two types of intervention, disregarding the caloric intake of 100 g of cereal product per day that was equivalent for both products (Table S1).

### MF intervention reduced intestinal inflammation but not metabolic endotoxemia nor systemic inflammation

**Figure 2.**
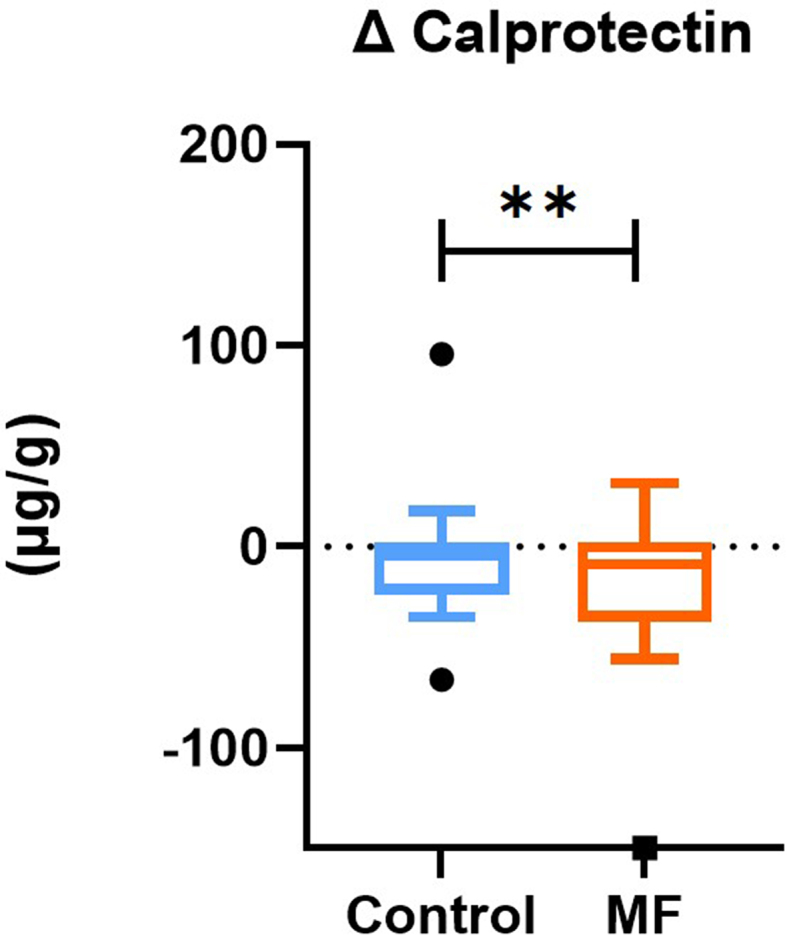
**MF intervention has reduced Calprotectin compared to control.** Fecal fasting calprotectin was measured before and after 8-week treatment in at-risk subjects (n=30). Tukey box-plots (10-90 percentile) display the delta between the end and the beginning of the intervention (8 weeks); with blue lines refer to subjects who received the control intervention; orange lines refer to those who received MF intervention. A linear mixed model for repeated measures, with a compound symmetry structure as the covariance structure after Blom transformation, was used to determine whether the difference between the changes induced by 8-week MF intervention compared to Control were statistically significant. **p < 0.01.

**Figure 3.**
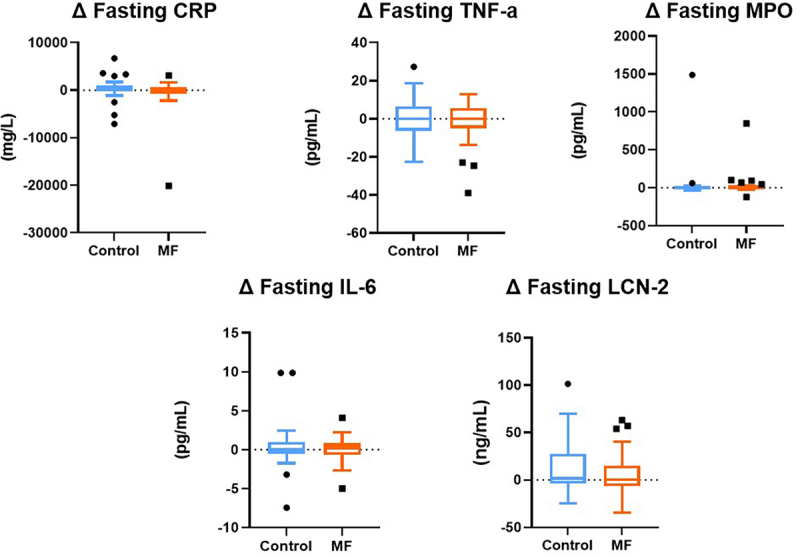
**MF intervention has not significantly altered fasting systemic inflammation or cardiovascular-risk markers compared to control.** Low-grade inflammation and cardiovascular risk markers were not significantly impacted by multifunctional intervention (n=30) compared to control.  Tukey box-plots (10-90 percentile) display the delta between before and after intervention (8 weeks); with blue lines refer to subjects who received the control intervention; orange lines refer to those who received MF intervention. A linear mixed model for repeated measures, with a compound symmetry structure as the covariance structure after Blom transformation, was used to determine whether the difference between the changes induced by 8-week MF intervention compared to Control were statistically significant.

Compared to Control, the 8-week MF intervention induced a significant reduction in fecal calprotectin concentrations, marker of intestinal inflammation (*p* = 0.007, Figure 2). Regarding endotoxemia, MF intervention did not significantly change either fasting or postprandial excursions of LPS, LBP, sCD14, or LPB/sCD14 (Table 2). We did not observe any significant impact on fasting (Figure 3 and Table 2) and postprandial (data not shown) systemic inflammation and cardiovascular risk markers.

**Table 2.** Effects of 8 weeks of MF intervention compared to control intervention on inflammation parameters (*n* = 30).

	MF intervention						Control intervention		
	Baseline		After 8 weeks		Baseline		After 8 weeks		p-value (time*group)
Endoxotemia parameters									
tAUC LBP/sCD14 ratio	3008	(1588)	2725	(1410)	2922	(1485)	2924	(992)	0.37
tAUC LBP (µg/mL*min)	3508	(1776)	3651	(1391)	3506	(1026)	3626	(1664)	0.62
tAUC sCD14 (µg/mL*min)	376	(76)	394	(116)	368	(102)	378	(104)	0.68
LBP (µg/mL)	12.1	(7.3)	12.9	(5.2)	10.8	(5)	12.8	(5.8)	0.52
sCD14 (µg/mL)	1.2	(0.4)	1.4	(0.4)	1.3	(0.3)	1.3	(0.4)	0.31
LPS (ng/mL)	28.5	(33.8)	28.5	(33.1)	25.9	(31.3)	26.3	(24.5)	0.78
tAUC_LPS	9783	(8077)	10614	(8804)	9332	(9184)	9334	(9618)	0.18
Inflammation/CV risk parameters									
CRP (µg/L)	1155	(3116)	1474	(2269)	1019	(3178)	1558	(2784)	0.53
MCP-1 (pg/mL)	34	(16.9)	38.2	(38.2)	37.5	(12.1)	34.9	(16.4)	0.87
IFN-g (pg/mL)	3.6	(2.1)	3.2	(1.9)	4	(4.5)	3.5	(2.2)	0.14
IL-6 (pg/mL)	2.5	(2.4)	2.2	(1.2)	2.2	(1.9)	2.4	(2)	0.87
TNF-a (pg/mL)	19.3	(10.7)	21.1	(8.7)	19.3	(6.5)	22.5	(16.3)	0.92
ADAMTS13 (ng/mL)	1887	(914)	1839	(722)	1888	(674)	2033	(758)	0.48
Myeloperoxydase (pg/mL)	43.7	(20.4)	40.4	(27.8)	35.9	(19.9)	40.6	(21.3)	0.90
Lipocalin2 (ng/mL)	126	(38)	133.3	(71)	126	(45)	135	(56)	0.26
sVCAM1 (ng/mL)	1048	(290)	1092	(419)	1045	(176)	1083	(352)	0.1
SAA (ng/mL)	8146	(11524)	9100	(8046)	9100	(7071)	8960	(6621)	0.07

Data are expressed as median and IQR (interquartile range). Effects of MF intervention compared to Control were analyzed using linear mixed model for repeated measures with heterogeneous compound symmetry as covariance structure after Blom transformation, with associated p-value from the interaction time*group. CV: cardiovascular; tAUC: total area under the curve; LBP: lipopolysaccharide binding protein; sCD14: soluble cluster of differentiation 14; LPS: lipopolysaccharide; CRP: C-reactive protein; MCP-1: monocyte chemoattractant protein 1; IFN-g: Interferon gamma; IL-6: Interleukin 6; TNF-a: tumor necrosis factor α; ADAMTS13: a disintegrin and metalloprotease with thrombospondin type I repeats-13; sVCAM1: Soluble Vascular Cell Adhesion Molecule-1; SAA: Serum amyloid A.

### MF intervention reduced fasting endotoxemia after 1-week fructose challenge

**Figure 4.**
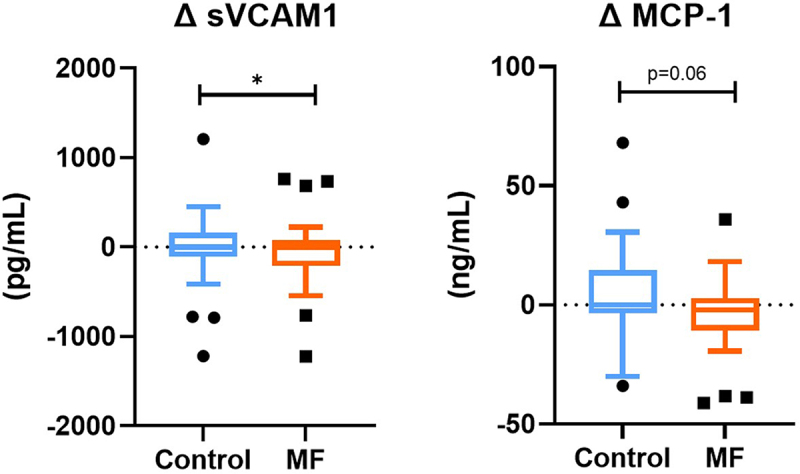
**MF intervention reduces fructose-induced inflammation compared to control**. Cardiovascular-risk markers were significantly reduced by multifunctional intervention (n=30) compared to control between after and before 1-week fructose challenge (delta between week 9 and 8). Tukey box-plots (10-90 percentile) with blue lines refer to subjects who received the control intervention; orange lines refer to those who received MF intervention. A linear mixed model for repeated measures, with a compound symmetry structure as the covariance structure after Blom transformation, was used to determine whether the difference between the changes induced by MF intervention compared to Control were statistically significant. *p < 0.05.

8-week MF intervention reduced the fructose-induced increase in fasting LPS compared to the Control intervention (*p* = 0.03, Table 3), with no impact on incremental postprandial LPS response. Similarly, after MF intervention compared to Control, a reduction in soluble vascular cell adhesion molecule 1 (sVCAM1) levels (*p* = 0.01) and a trend toward reduced levels of monocyte chemoattractant protein 1 (MCP-1) (*p* = 0.06) were observed following the fructose challenge (Figure 4).

**Table 3.** Effects of 1-week fructose challenge on inflammation parameters (*n* = 30).

	MF intervention				Control intervention				
	9 weeks		Δ before and after fructose week		9 weeks		Δ before and after fructose week		p-value (time*group)
Fasting inflammation parameters									
tAUC LBP/sCD14 ratio	2595	(1301)	−130	(873)	2788	(1268)	−136	(1046)	0.47
tAUC LBP (µg/mL*min)	3330	(1659)	−321	(656)	3481	(1570)	−145	(1018)	0.65
tAUC sCD14 (µg/mL*min)	398	(123)	4.0	(118)	391	(137)	13	(101)	0.48
LBP (µg/mL)	12.6	(5.4)	−0.3	(3.5)	12.1	(7.3)	−0.7	(5.8)	0.53
sCD14 (µg/mL)	1.4	(0.5)	0	(0.31)	1.2	(0.5)	−0.1	(0.26)	0.51
LPS (ng/mL)	24.3	(30.3)	−4.2	(14.4)	27.7	(39.6)	1.4	(19.9)	0.03
tAUC LPS	8580	(9518)	−2034	(4635)	8632	(8664)	−702	(3313)	0.19
Inflammation/CV risk parameters									
CRP (µg/L)	1626	(2636)	152	(770)	1548	(2190)	−10	(1347)	0.99
IFN-g (pg/mL)	3.4	(4.2)	0.2	(4.6)	3.2	(3.8)	−0.3	(5.2)	0.25
IL-6 (pg/mL)	2.3	(2.3)	0.1	(1.1)	2.1	(1.4)	−0.3	(1.5)	0.18
TNF-a (pg/mL)	18	(11.9)	−3.1	(10.3)	21.7	(13.1)	−0.8	(9.6)	0.37
ADAMTS13 (ng/mL)	1798	(789)	−41	(625)	1992	(555)	−41	(762)	0.26
Myeloperoxydase (pg/mL)	36.4	(18.1)	−4.0	(24.8)	34.1	(19.1)	−6.5	(12.1)	0.79
Lipocalin2 (ng/mL)	128	(43)	−4.0	(30)	131	(41)	−4.0	(34)	0.14
SAA (ng/mL)	7467	(5844)	−1633	(3672)	8165	(9829)	−795	(2467)	0.28

Data are expressed as median and IQR (interquartile range). Effects of MF intervention compared to Control were analyzed using linear mixed model for repeated measures with heterogeneous compound symmetry as covariance structure after Blom transformation, with associated p-value from the interaction time*group. CV: cardiovascular; tAUC: total area under the curve; LBP: lipopolysaccharide binding protein; sCD14: soluble cluster of differentiation 14; LPS: lipopolysaccharides; CRP: C-reactive protein; MCP-1: monocyte chemoattractant protein 1; IFN-g: Interferon gamma; IL-6: Interleukin 6; TNF-a: tumor necrosis factor α; ADAMTS13: a disintegrin and metalloprotease with thrombospondin type I repeats-13; sVCAM1: Soluble Vascular Cell Adhesion Molecule-1; SAA: Serum amyloid A.

**Figure 5.**
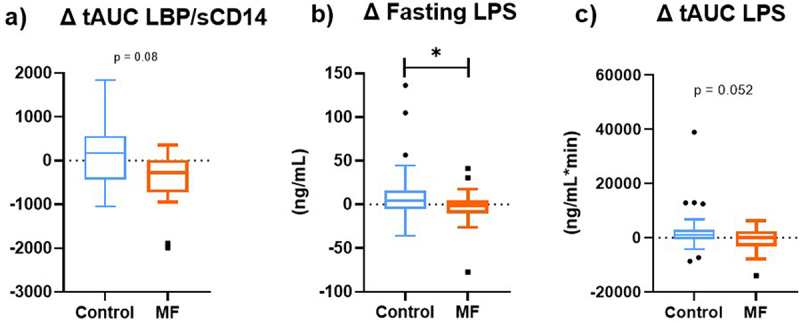
**MF intervention tend to reduce postprandial LBP/sCD14 excursions and reduce fasting endotoxemia (LPS) compared to control after 1-week fructose challenge. a)** Post-prandial (total AUC between 0, 120 and 300 min) plasma LBP/sCD14 was measured in at-risk subjects (n=30) between the pre-intervention and 9-week periods. **b)** Fasting plasma LPS (ng/mL) was measured between the pre-intervention and 9-week periods. **c)** Post-prandial (total AUC between 0, 120 and 300 min) of LPS concentrations during the MF and control interventions between the pre-intervention and 9-week periods after ingestion of the standardized meal . Box-plot display the delta between the end of the intervention (after 8-week +1-week fructose) and before intervention. Tukey box-plots (10-90 percentile) with blue lines refer to subjects who received the control intervention; orange lines refer to those who received MF intervention. A linear mixed model for repeated measures, with a compound symmetry structure as the covariance structure after Blom transformation, was used to determine whether the difference between the changes induced by 9-week MF intervention compared to Control were statistically significant. *p < 0.05.

Altogether, after 8 weeks of MF intervention and 1 week of fructose challenge, postprandial excursions total Area Under the Curve (tAUC) of LBP/sCD14 tended to be lower than baseline, compared to Control (−10.3% versus + 0.07% for Control Figure 5a, *p* = 0.08), with no parallel effect on fasting LBP/sCD14 ratio. MF also induced a reduction of LPS in the fasting state (Figure 5b) and a trend for postprandial (tAUC) reduction after 8 weeks of intervention +1 week of fructose challenge versus baseline compared with Control (*p* < 0.05 and *p* = 0.52 respectively, Figure 5c).

### The MF intervention modified gut microbiota composition

**Figure 6.**
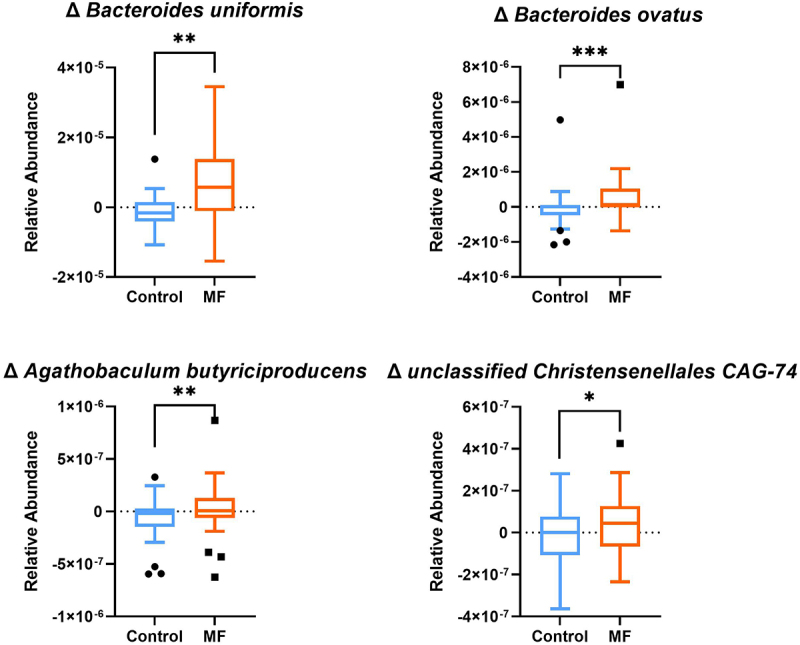
**MF intervention have increased beneficial gut microbiota species relative abundance (MSP) compared to control**. Gut microbiota abundance was significantly impacted by multifunctional intervention (n=30). Histograms display the delta between the end of the intervention (after 8 weeks intervention) and before intervention. Tukey box-plots (10-90 percentile) with blue lines refer to subjects who received the control intervention; orange lines refer to those who received MF intervention. MSP: Metagenomic Species; p-values from Wilcoxon signed-rank test are displayed. *p < 0.05 **p <0.01 ***p <0.001

After 8 weeks, compared to Control, MF intervention brought about changes in the composition of gut microbiota by adjusting the prevalence of specific bacterial species (Figure 6). Among 391 tested species (prevalence >10%), 27 were significantly impacted by the MF intervention (Figure S2, p&lt;0.05). Specifically, metagenomics shotgun analysis showed a significant increase in the relative abundance of *Bacteroides ovatus* (*p* = 0.0049), *Bacteroides uniformis* (*p* = 0.0006), *Agathobaculum butyriciproducens* (*p* = 0.005) and a non-significant modulation of *Akkermansia muciniphila* (*p* = 0.1, data not shown), compared to a decrease after the Control intervention. Throughout the dietary intervention, microbiota richness in terms of gene count and Metagenomic Species (MSPs). MSP count did not differ significantly between the two interventions (Figure S3).

### MF intervention reduced serum branched chain amino acids and glutamate concentrations

**Figure 7.**
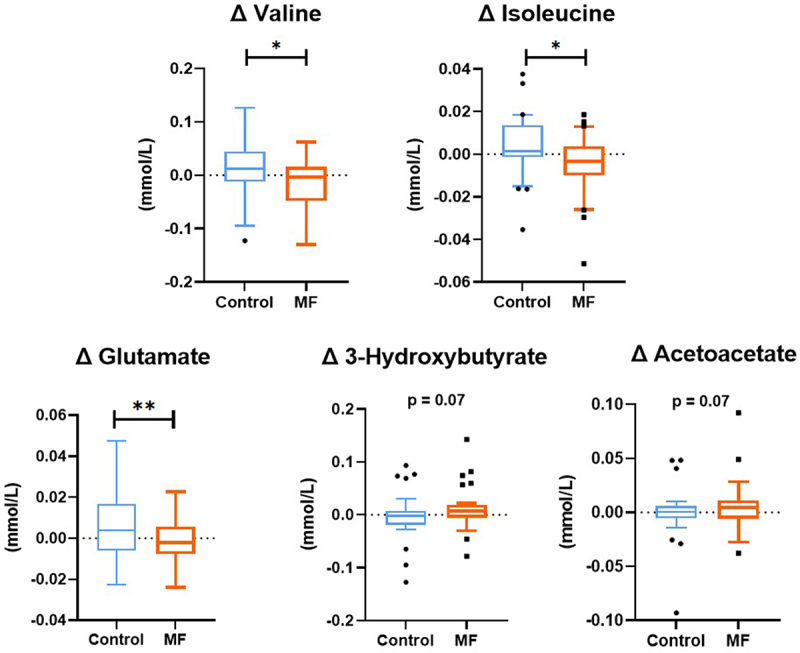
**MF intervention has modulated metabolomic profile evaluated by NMR technique (n=30)**. Box-plot display the delta between the end of the intervention (after 8 weeks intervention) and before intervention. Tukey box-plots with blue lines refer to subjects who received the control intervention; orange lines refer to those who received MF intervention. A linear mixed model for repeated measures, with a compound symmetry structure as the covariance structure after Blom transformation, was used to determine whether the difference between the changes induced by 8-week MF intervention compared to Control were statistically significant. *p < 0.05 **p <0.01.

Quantitative metabolomic analysis of 40 serum metabolites by proton nuclear magnetic resonance spectroscopy^1^H-NMR, Table S2) showed a significant decrease of 2 branched chain amino acids (BCAAs) namely valine and isoleucine after 8 weeks of intervention (−5% and −7% after MF respectively, *p* < 0.05) and a trend for acetoacetate and 3-hydroxybutyrate, compared to Control intervention. These analyses also showed a significant decrease in serum glutamate levels after 8 weeks of MF intervention vs control (*p* < 0.05, Figure 7).

### MF intervention did not alter anthropometric nor metabolic markers but tended to decrease adipo-ir

Eight weeks of intervention with MF did not result in any significant change in weight or Body Mass Index compared to control (Table 4). In addition, no effect of either MF or control was observed in body composition, or in percentages of fat free mass and of fat mass. Fasting or postprandial metabolic parameters remained similar after both MF and Control interventions (Table 4). However, MF intervention compared to the control intervention tended to decrease adipo-IR insulin resistance index (*p* = 0.08).

**Table 4.** Effects of 8 weeks of MF intervention compared to control intervention on metabolic parameters (*n* = 30).

	MF Intervention				Control intervention				
	Baseline		After 8 weeks		Baseline		After 8 weeks		p-value (time*group)
Anthropometric parameters									
Weight (kg)	83.8	(16.5)	83.8	(16.5)	84.1	(16.8)	85.1	(16.9)	0.36
BMI (kg/m^2^	29.0	(3.2)	29.0	(3.6)	28.9	(3.1)	29.0	(3.4)	0.81
Fat mass (kg)	30.6	(8.1)	31.5	(8.8)	30.8	(8.7)	30.6	(9.2)	0.85
Fasting metabolic parameters									
Glucose (mM)	5.3	(0.5)	5.3	(0.5)	5.4	(0.6)	5.2	(0.5)	0.76
NEFA (µM)	462	(244)	475	(250)	443	(170)	472	(176)	0.28
Insulin (mIU/l)	10.6	(3.8)	9.3	(6)	12.1	(4.6)	11.4	(8.7)	0.33
HOMA-IR	2.4	(1)	2.2	(1.6)	2.8	(1.2)	2.7	(2.6)	0.23
Adipo-IR	39.1	(17)	35.5	(15.6)	40.2	(23.8)	40.4	(30.6)	0.08
Total cholesterol (mM)	4.8	(1.1)	4.7	(1.2)	4.8	(0.9)	4.6	(1.3)	0.64
HDL cholesterol (mM)	1.1	(0.3)	1.1	(0.3)	1.2	(0.3)	1.2	(0.3)	0.15
LDL cholesterol (mM)	3.2	(0.7)	3.0	(1.1)	3.1	(0.9)	3.1	(1.2)	0.76
ApoB (g/L)	0.9	(0.28)	0.9	(0.3)	0.9	(0.3)	0.9	(0.3)	0.35
Adiponectin (μg/mL)	6.3	(2.9)	5.1	(2.9)	4.9	(2.9)	5.4	(3.2)	0.49
Postprandial metabolic parameters									
Glucose tAUC (mM*min)	1754	(228)	1783	(160)	1720	(173)	1789	(253)	0.28
Insulin tAUC (mUI/l*min)	13583	(4908)	11143	(4933)	12273	(7292)	12850	(8839)	0.21
TG tAUC (mM*min)	556	(429)	568	(457)	542	(298)	566	(350)	0.98

Data are expressed as median and IQR (interquartile range). Effects of MF intervention compared to Control were analyzed using linear mixed model for repeated measures with heterogeneous compound symmetry as covariance structure after Blom transformation, with associated p-value from the interaction time*group. MF: multifunctional intervention; BMI: body mass index; NEFA: non-esterified fatty acid; HOMA-IR: homeostasis model assessment of insulin resistance; Adipo-I.0 R: Adipose tissue insulin resistance; HDL cholesterol: high-density lipoprotein cholesterol; LDL cholesterol: low-density lipoprotein cholesterol; ApoB: Apolipoprotein B; TG: triglyceride; tAUC: total area under the curve.

The fructose challenge did not significantly alter fasting or postprandial metabolic parameters related to carbohydrate or lipid metabolism after MF or Control intervention (Table 5).

**Table 5.** Effects of 1-week fructose challenge after MF or control intervention on metabolic parameters (*n* = 30).

	MF intervention				Control intervention				
	After 1 week of fructose challenge		Δ before and after fructose week		After 1 week of fructose challenge		Δ before and after fructose week		p-value (time*group)
Fasting metabolic parameters									
Glucose (mM)	5.4	(0.3)	0.1	(0.3)	5.4	(0.5)	0.2	(0.3)	0.80
NEFA (µM)	471	(207)	−4.0	(237)	443	(149)	−29.0	(203)	0.77
Insulin (mIU/l)	11.9	(7.3)	2.6	(4.1)	12.1	(8)	0.7	(4)	0.33
HOMA-IR	2.8	(2.3)	0.6	(1.1)	2.9	(2.4)	0.2	(1.1)	0.41
Adipo-IR	33.4	(26.9)	−2.1	(17.7)	37.9	(27.6)	−2.5	(18.8)	0.62
Total cholesterol (mM)	4.5	(1.5)	−0.2	(0.8)	4.8	(1.5)	0.2	(0.8)	0.94
HDL cholesterol (mM)	1	(0.3)	−0.1	(0.2)	1.1	(0.3)	−0.1	(0.1)	0.47
LDL cholesterol (mM)	2.6	(1)	−0.4	(0.7)	3	(1.3)	−0.1	(0.8)	0.62
ApoB (g/L)	0.8	(0.3)	−0.1	(0.1)	0.9	(0.4)	0	(0.2)	0.63
Adiponectin (μg/mL)	5.3	(3.8)	0.2	(2.4)	5	(3)	−0.4	(1.7)	0.60
Postprandial metabolic parameters									
Glucose tAUC (mM*min)	1759	(287)	−24	(169)	1758	(215)	−31	(123)	0.81
Insulin tAUC (mUI/l*min)	13786	(7280)	2643	(4357)	13781	(5402)	931	(3806)	0.17
TG tAUC (mM*min)	694	(429)	126	(361)	708	(439)	142	(189)	0.62

Data are expressed as median and IQR (interquartile range). Effects of MF intervention compared to Control were analyzed using linear mixed model for repeated measures with heterogeneous compound symmetry as covariance structure after Blom transformation, with associated p-value from the interaction time*group. MF: multifunctional intervention; BMI: body mass index; CV: cardiovascular; NEFA: non-esterified fatty acid; HOMA-IR: homeostasis model assessment of insulin resistance; Adipo-IR: Adipose tissue insulin resistance; HDL cholesterol: high-density lipoprotein cholesterol; LDL cholesterol: low-density lipoprotein cholesterol; ApoB: Apolipoprotein B; TG: triglyceride; tAUC: total area under the curve.

### Association between mf-induced changes in endotoxemia, fecal calprotectin, gut microbiota and metabolites

**Figure 8.**
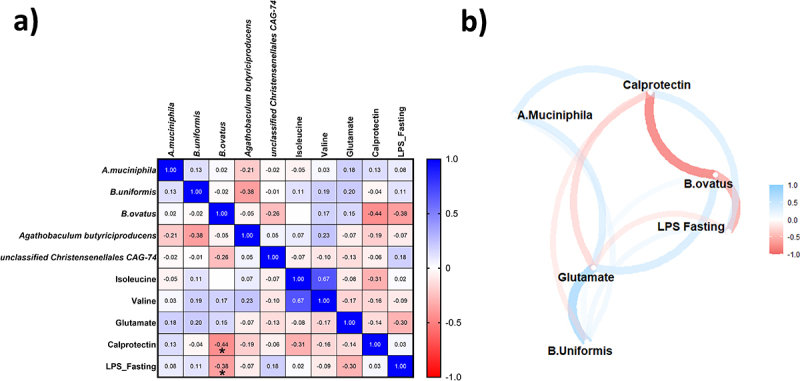
(a) Spearman’s rank correlation matrix between parameters significantly impacted by after MF intervention compared to control (b) A correlation network map depicting the Spearman’s correlation matrix was generated. Metabolites exhibiting high correlation were clustered together. The arrangement of metabolites was determined through multidimensional scaling of the absolute correlation values. Gradient color, line distance, and thickness were assigned to metabolite nodes based on correlation coefficients. Negative and positive correlations were represented by shades of red and blue, respectively. Stars (*) stand for correlations that are significant regarding p-values (p < 0.05).

We correlated the delta (after – before the MF intervention) of significantly impacted metagenomics features, inflammatory markers (fasting LPS and calprotectin) and key amino acids quantified by 1 h-NMR (Figure 8). Spearman correlation analyses showed that changes in *Bacteroides ovatus* abundance was negatively correlated with fasting LPS (*r* = −0.38 *p* < 0.05) and fecal calprotectin levels (*r* = −0.44, *p* < 0.05).

## Discussion

Our study reveals that an 8-week multifunctional dietary intervention with a concept product combining selected bioactive compounds in individuals with cardiometabolic risk factors improves intestinal inflammatory profile and induces a significant decrease in serum branched-chain amino acids, while modulating several bacterial species of the gut microbiota.

Regarding intestinal inflammatory profile, we show that altogether substituting usual consumption of regular cereal product with multifunctional bioactive enriched-Cereal Product reduced calprotectin and improved endotoxemia profile. Specifically, MF intervention tended to lower postprandial metabolic endotoxemia as shown with LPS and LBP/sCD14 markers and reduced fasting LPS compared to control. Metabolic endotoxemia can be due to an increased intestinal permeability allowing the passage of LPS to the bloodstream via the para-cellular path, which is now referred to as “leaky gut”.^31^ Furthermore, such LPS translocation can also occur via the transcellular pathway during lipid absorption involving chylomicrons as LPS carriers.^32^ Leveraging the postprandial phase, we demonstrated a promising trend toward improved metabolic endotoxemia, as indicated by the markers LBP/sCD14 and LPS. Improvements of endotoxemia markers have also been observed elsewhere with integrated diets such as the Mediterranean diet but also using bioactive compounds tested individually such as omega-3 lipids or polyphenols but also following the administration of *A. muciniphila* in subjects with CM risks.^21,33–35^ Flavonoids, which represents the family of polyphenols tested in the present MF, intervention and their metabolites have been shown to possess favorable properties in modulating the gut environment by decreasing circulating LPS levels and by reshaping the gut microbiota.^36^ In the present study, we illustrate the beneficial impact of the MF intervention on several markers of endotoxemia, both at the fasting and postprandial states (trend), which may result from reduced intestinal permeability and limited LPS translocation during lipid ingestion compared to the control.

In addition, MF intervention significantly lowered calprotectin (−40%), a protein released by neutrophils as part of the inflammatory response. This marker is traditionally examined in clinical settings to assess levels of intestinal inflammation and has been investigated widely in the context of chronic intestinal disease such as inflammatory bowel disease.^37,38^ However, fecal calprotectin remains poorly studied in the context of nutritional interventions targeting cardiometabolic profile.^39^ Importantly, the calprotectin reduction observed here suggests an improvement in the intestinal inflammation profile, bringing it below the 50 µg/g threshold that is consistent with inflammation levels typically found in healthy individuals.^40^ Interestingly, in preclinical studies, certain dietary bioactive compounds such as polyphenols and omega-3 have shown beneficial effects on inflammation and intestinal permeability.^41,42^ Additionally, dietary fibers, particularly soluble fibers, are known to produce short-chain fatty acids (SCFAs) through fermentation, which have beneficial effects on intestinal health. Specifically, these SCFAs, notably butyrate, is known to contribute to the integrity of the intestinal epithelium, the inhibition of intestinal inflammation, and the maintenance of tight junctions.^43^

The present results reinforce the potential protective efficacy of combining several bioactive compounds within a food product on intestinal inflammation, echoing the findings observed on fasting metabolic endotoxemia.

We observed a relevant effect on inflammatory and cardiovascular risk, highlighted by the fructose challenge after MF intervention compared to control. The MF intervention notably induced a protective effect against the fructose challenge by reducing sVCAM1 and a trend for MCP-1, both recognized as pivotal players at the intersection of inflammation and cardiovascular risk. MCP-1, a chemokine implicated in insulin resistance and vascular dysfunction, and sVCAM1, regarded as a cardiovascular disease predictor, were positively modulated following the intervention.

Through this challenge, we demonstrated the early detection of the impact of MF intervention in counteracting inflammation induced by a one-week fructose challenge, consistent with the findings of Hokayem et al. regarding grape polyphenols intervention.^44^ This validates the use of such a challenge in subjects at cardiometabolic risk, highlighting a high quantity of fructose as a physiological stressor that enhances the detection and quantification of dietary effects on the multi-component inflammatory state.

Interestingly, as found in our study, consumption of polyphenols from the Mediterranean diet has shown a reduction in markers associated with atherosclerosis such as MCP-1 and sVCAM-1 in the PREDIMED trial.^45^ This demonstrates a convergent impact of MF intervention on an array of different intestinal and systemic inflammation markers. The different amplitude of effects between intestinal and circulating markers may stem from their connection to distinct physiological pathways, systemic inflammation markers being more largely indicative of the interconnection among various local inflammatory pathways associated with organs and systemic processes. We have recently reviewed such discrepancies in different inflammatory markers in responses to dietary MF interventions, independently of the chosen compound combinations, highlighting the importance of analyzing a wide range of markers, in particular inflammatory endpoints, for the local and systemic metabolic impact of diet.^10^

Here we also demonstrate that a multi-target approach, using dedicated challenge tests, allows to investigate the dynamic impact of diet on the cascade of mechanisms involved in low-grade inflammation, considering the role of endotoxemia and gut integrity, which act as a link between gut microbiota and inflammatory markers.^36^

We hypothesized that these favorable findings potentially stem from the convergent influence of the selected bioactive compounds on the modulation of inflammation and on specific bacterial taxa known to exert immunomodulatory effects. The MF intervention changed the composition of the intestinal microbiota and notably certain species well known for their beneficial role on inflammatory and metabolic parameters. After MF, *Bacteroides ovatus* was increased, and was negatively associated with metabolic endotoxemia and intestinal inflammation. This bacterium has been demonstrated to support intestinal balance by preserving the diversity of the gut microbiota and reducing LPS-induced inflammation by reinstating the equilibrium between Treg and Th-17 cells (known to regulate the balance between pro- and anti-inflammatory pathways).^46^ Furthermore, *Bacteroides uniformis* which was increased in our study, improves metabolic and immune dysfunctions induced by an obesogenic diet and concomitant with gut microbiota deviation in obese mice.^47^ Interestingly, following MF intervention, the relative abundance of an unclassified *Christensenellaceae* species increased compared to control and have been shown to be inversely correlated with the individual BMI across several investigations as reviewed by Waters et al.^48^

In addition, alpha-linolenic acid (ALA) from rapeseed oil may also contribute to some of the observed benefits, as it has been shown to promote the abundance of these beneficial bacteria compared to the presence of palmitic acid in control which has been associated with altered gut integrity and increased inflammation.^49–51^

The analysis of the serum metabolome notably showed a decrease in certain BCAA, mainly valine and isoleucine upon MF intakes. Several studies have indicated that plasma BCAA levels are elevated in overweight and obese individuals and correlated with insulin resistance, as well as in T2D patients.^52,53^ In addition, we found a trend toward increased levels of certain compounds involved in ketone body metabolism, notably acetoacetate and 3-hydroxybutyrate, phenomenon that may reflect the catabolism of BCAA (Supplementary Figure S1). Moreover, the gut microbiota can produce BCAA that can be modulated by dietary supplementation of bioactive compounds.^54^ Indeed, supplementation with polyphenols effectively mitigated the increase of BCAAs induced by an overnutrition regimen among healthy, overweight participants.^55^ In the PREDIMED trial, a Mediterranean diet significantly decreased BCAA levels and weakened the link between plasma BCAAs and type 2 diabetes incidence.^56^ These beneficial changes in BCAA profile are supported by a trend toward improvement in adipo-IR, a marker of adipose tissue insulin resistance. Moreover, our study demonstrates a significant reduction in serum glutamate concentrations after the 8-week MF intervention compared to the Control group. This can be considered as beneficial because elevated levels of glutamate have been linked to a heightened incidence of type 2 diabetes, cardiovascular diseases, and nonalcoholic fatty liver disease.^57,58^

Of note, the obtained favorable metabolic effects were observed without modification of food intake and body weight loss during the study, parameters known to be closely linked with an improved CM risk profile.^59,60^

The present investigation possesses numerous strengths: 1) Van den Brinck et al suggest that inflammatory conditions in at-risk individuals stem from a cascade of events disrupting homeostasis.^2^ Our study assesses phenotypic flexibility and postprandial food dynamics to detect early metabolic disturbances; 2) this study adopts a randomized crossover design, effectively mitigating the risk of false-positive associations, as participants serve as their own controls; 3) we conducted an integrative analysis of multi-level health markers, establishing connections between gut health, systemic modulations, and inflammatory mediators such as endotoxin; 4) this study aimed to capture the dynamics involved in the development of low-grade inflammation by linking different actors such as cytokines, adipokines and proteins mediated by different tissues 5) furthermore, considering the high compliance observed, this study encourages further research in longer-term trials. To standardize and control bioactive compounds’ intake, intervention was delivered through enriched validated cereal product by replacement of usual consumed cereal product.^30^

Limitations of the study include the fact that the target population had a high waist circumference but was heterogeneous in terms of metabolic profile, leading to variability in metabolic responses and a limited population size. Additionally, to address the leaky gut hypothesis, measuring zonulin, a key biomarker of intestinal permeability, would have provided valuable insights into gut barrier function, potentially revealing whether the intervention impacted intestinal permeability, a factor closely linked to metabolic disorders and systemic inflammation. Moreover, the potential insights into the functionality of the intestinal microbiota remained untapped due to the limited sequencing depth. This valuable information could have provided a more nuanced understanding of the role of bacteria in the subject responses. Given the multiplicity of criteria, alpha risk inflation makes subgroup analysis complex; however, it would have been valuable to identify the determinants of response to the intervention by conducting subgroup analyses of responders and non-responders based on baseline characteristics. Finally, the promising results regarding all exploratory parameters of this proof-of-concept will need further confirmation in follow-up clinical trials.

In conclusion, a 2-month multifunctional dietary intervention was efficient to improve intestinal inflammatory profile and modulate gut microbiota composition including changes in beneficial microbes. These results, combined with the high level of compliance of product intake among the participants, indicate the potential of MF dietary approaches with multi-target impact as effective strategies to target intestinal inflammation and metabolic health in overweight and obese subjects with CM risk.

## Materials and methods

### Clinical study

The SINFONI study was conducted at the Human Nutrition Research Center Rhône-Alpes (CRNH-RA) and carried out in accordance with the Second Declaration of Helsinki and French Jardé law. The study was reported and registered on http://www.clinicaltrials.gov (NCT04190706) and was approved by the Scientific Ethics Committee of Bordeaux Sud-Ouest and Outre-Mer III. All participants received and signed informed consent before the initiation of any study-related procedure.

### Study design

**Figure 9.**
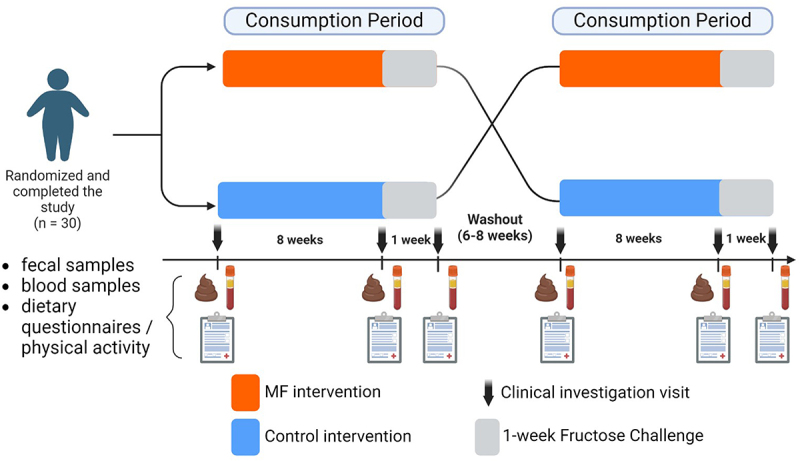
SINFONI Experimental design. In a randomized crossover study, participants were selected to assess the impact of MF intake on metabolic, inflammatory and gut microbiota parameters. Each participant underwent both the MF intervention and a control intervention in a randomized order, with a 6 to 8-week washout period between interventions to minimize carryover effects.

This was single center, randomized, single blind, crossover comparison intervention study with two sequences and a 6-to-8-week washout period (Figure 9). The two dietary interventions consisted of 100 g/d of cereal-based product enriched with bioactive products (Multifunctional, MF) and 100 g/d of Control cereal-based product products that could be consumed at any time of the day. At the end of each intervention arm, metabolic and inflammatory markers were re-assessed after a metabolic challenge consisting of one-week fructose supplementation (3 g/kg fat-free mass/day), ingested orally as a powdered form dissolved in the volunteers’ daily drinking water.^44^ The fructose challenge was implemented to evaluate whether the 8-week MF intervention could modulate the response to pro-oxidative, pro-inflammatory challenge.

### Study participants

Thirty individuals either overweight or with obesity and at-CM risk (50% women) were included with the following eligibility criteria: age 30–65 years, BMI between 25 and 35 kg/m^2^ and waist circumference >80 cm for women and >94 cm for men, with a stable weight over the last 3 months (<5% of total body weight), and regularly snacking with cereal products in order to substitute this consumption with the study products. We included subjects who were sedentary or engaged in a stable regular physical activity (maximum 4 h per week); subjects with total cholesterol <11 mmol/L or triglycerides <3 mmol/L, fasting blood glucose <7 mmol/L, CRP <20 mg/L and no biological abnormalities deemed clinically significant by the investigator. Subjects were not on a restrictive diet and were selected based on a low consumption of fiber (<25 g/day) and polyphenols (less than 3 coffees + 1 tea per day). The study included only participants who did not consume dietary supplements (fiber, pre or probiotics) or small fruits (100 g) such as berries or their derivatives, as well as polyphenols (assessed during the dietary interview). We excluded subjects with cardiometabolic diseases such as diabetes, gastrointestinal anomaly or any untreated and unstable pathology. In addition, subjects should not have received antibiotics or other medications interfering with microbiota composition within the last 3 months prior to and during the study.

### Study products and diet intervention

We have previously formulated and validated MF cereal-based products enriched in selected bioactive compounds.^30^ Briefly, the two enriched cereal products were nutritionally improved with enrichments in fibers and polyphenols and with maintaining high-SDS content and improving fat quality using rapeseed oil, compared to control biscuits in which palm oil was incorporated (Table 6). The MF products contained more than 1% of total polyphenols (g eq gallic acid/100 g, current daily dose consumed in France <1 g/d) from cranberries containing mostly tannins notably proanthocyanidins (Symrise, Germany) and 18 g of dietary fibers (cranberry) allowing to reach >30 g/day, a dose recommended by the WHO.^30,61,62^ Moreover, 100% of lipids in the products are supplemented using rapeseed oil to increase alpha-linolenic acid quantity, a type of omega 3. The difference in fatty acid composition between the MF and Control cereal products) is provided by the presence of rapeseed oil for the MF product (12% with 1.5 g of omega 3) and palm oil for the Control product (14%, rich in saturated fatty acids). We used specific cooking processes to reach 33% and 40% of slow digestible starch (SDS) in MF products that is twice the quantity within the Control products.

**Table 6.** Nutritional composition of biscuits.

	Control mini-biscuits	Control cookies	MF mini-biscuits	MF cookies
Energy (Kcal/100 g)	472	480	439	430
Lipids (g/100 g)	17.5	20.6	18.1	17
−Saturated	9	10	1	1
-Polyunsaturated *n*-3	0	0	2	2
Proteins (g/100 g)	6.6	5.1	7.4	6.6
Available carbohydrates	71.4	64.7	53.5	53.5
(g/100 g)				
Sugars (g/100 g)	28.9	29.6	18.9	20.5
Available starch	39.8	30.7	27.9	24.5
(g/100 g)				
SDS (g/100 g)	7.8	4.9	12.8	10.2
Total dietary fibers (g/portion)	2	2	18	19
Insoluble dietary fibers (g/100 g)	1	1	15	16
Soluble dietary fibers (g/100 g)	1	1	3	3
Total polyphenols (g eq gallic acid/100 g)	0.6	0.2	2.6	2.2

The food product prototypes were produced in the pilot plan of Mondelez International R&D Centre based in Saclay (France). Both interventions contained equal proportions of two types of products: cereals mini cookies or biscuits to obtain 100 g per day of food intake. The individuals were asked to replace their usual consumption of cereal-based biscuits during the day by the provided MF or Control cereal-based products.

### Dietary intake and compliance calculation

At inclusion, dietary intake of each subject was evaluated using the French National Authority for Health questionnaire together with an interview with a trained dietitian. Then, the subjects filled in a 3-day dietary record before exploration day to evaluate dietary intake and ensure good compliance. During the intervention periods, subjects were asked to maintain the same diet in terms of caloric and macronutrients content (out of provided intervention cereal-based products) and to fill-in a 3-day dietary record. Dietary data were processed by a trained dietitian using NUTRILOG^©^, which allowed to estimate energy, macronutrient and fiber intake. MF and Control products consumption compliance was evaluated by counting returned empty MF and Control packs. Subjects with less than 80% compliance were excluded from the study.

### Clinical investigation day

During the 3 days before metabolic assessment days, subjects were asked to collect a stool sample at ambient temperature using a specific kit containing RNAlater® as stabilizing solution, following the International Human Microbiome Standards (IHMS, SOP 05) () and send it to the French National Research Institute of Agriculture, Food and Environment (INRAE) MetaGenoPolis (mgps.eu) for analysis.

Subjects arrived at CRNH-RA after an 8-h overnight fast following the ingestion of a standard low dietary fiber evening meal (one serving of lean meat or fish, rice, a dairy product and fruit compote). Body weight, fat mass percentage, height and waist circumference were measured using a Bodystat Quadscan 4000 (BQ4000; Bodystat Ltd. Douglas, UK) stadiometer and non-elastic tape, respectively. BMI was calculated as weight/height.^2^ Blood was collected using an antecubital vein catheter at different times (T) (−30, 0, 15, 30, 60, 90, 120, 180, 240, 300 min). To evaluate postprandial metabolic response during each visit, the participants were also subjected to a “challenge test” meal (so-called FlexMeal) including the equivalent of an oral glucose tolerance test and a lipid load (semi-liquid meal based on the PhenFlex model,Table 7).^63,64^

**Table 7.** Nutritional composition of standardized test meal.

Energy (kcal)	917
Carbohydrates (g)	75.2
Proteins (g)	19.8
Lipids (g)	59.5
% carbohydrates	32.8
% proteins	8.6
% lipids	58.4
% MUFA	35.2
% PUFA	9.88
% SFA	48

MUFA: monounsaturated fatty acid; SFA: saturated fatty acid; PUFA: polyunsaturated fatty acid.

### Metabolic endotoxemia markers analyses

To calculate the ratio of LBP/sCD14 which has been proposed as a marker of proinflammatory potential of metabolic endotoxemia, LBP and sCD14 were assayed using sandwich ELISA kits (Hycult Biotechnology®, Quantikine® ELISA R&D Systems, respectively).^65^ Prior to analysis, plasma samples for LBP and sCD14 were diluted at 1/1000 and 1/400, respectively, as previously described.^65^ General LPS serotype O111:B4 ELISA kit was obtained from Biorbyt® (Cambridge, United Kingdom) on non-diluted plasma samples as a proxy of LPS passage in the bloodstream.^66^

### Inflammation parameters

The Millipore MILLIPLEX Human Cardiovascular Disease Magnetic Bead Panel 2 (SigmaAldrich®, France) was used to assess cardiovascular risk factor parameters, including measurements of Adamts13, MPO, Lipocalin-2/NGAL, sVCAM-1 and SAA. In addition, concentrations of the chemokines IL-6, INF-γ, TNF- α, MCP-1 were determined using the Bio-Plex Human Chemokines assay (Biorad®), following the manufacturer’s instructions. Quantitative determination of calprotectin in human feces was performed using the Calprotectin ELISA assay kit from Alpco®, USA. The stool samples were collected under real-life conditions by the volunteers using a designated collection kit. They were gathered within 48 hours before the metabolic exploration visit and were initially stored at −20°C by the participants. Upon arrival at the laboratory, the samples were transferred and stored at −80°C. Baseline CRP was measured with the IFCC and PP Architect Abott method, as previously described and with a human ELISA CRP kit from MyBioSource®, USA, was used to analyze blood samples during the study.^67^

### Biochemical blood analyses

Blood was collected and centrifuged immediately for 10 min at 4500 rpm at each exploration visit. Plasma was stored at *−*80*◦*C until the assays were conducted. Glycemia was measured by spectrophotometry according to Architect Abbott Hexokinase method; like triglyceride (TG), LDL-c, HDL-c, ApoB, ALAT, ASAT and CRP. Insulin was measured by electrochemiluminescence assay on Roche Cobas e411 system and non-esterified fatty acids (NEFA) by ACS-ACOD enzymatic assay with spectrophotometric measurement on INDIKO (Thermofisher®). Homeostasis Model Assessment of Insulin Resistance (HOMA-IR) was calculated as plasma glucose (mmol/L) x plasma insulin (mUI/L)/22.5. Adipo-IR (mmol × mIU/L) was calculated for each exploration visit by multiplying fasting NEFA concentrations (mmol/L) by fasting insulin (mIU/L) levels.

### Gut microbiota composition analysis

#### DNA extraction of stool samples and shotgun sequencing

Microbiota analysis was conducted either before or after the 8-week intervention. The extraction of DNA from fractions of fecal specimens was carried out following the MGP SOP 01 V1.^68^ Assessment of DNA quantity was done utilizing Qubit Fluorometric Quantitation (ThermoFisher Scientific, Waltham, US), and its quality was evaluated through DNA size profiling on a Fragment Analyzer (Agilent Technologies, Santa Clara, US). Library construction involved the use of three µg of high molecular weight DNA (>10 kbp). The DNA was fragmented to around 150 bp fragments employing an ultrasonicator (Covaris, Woburn, US), and library generation was executed utilizing the Ion Plus Fragment Library and Ion Xpress Barcode Adapters Kits (ThermoFisher Scientific, Waltham, US). Subsequently, purified and amplified DNA fragment libraries were subjected to sequencing using the Ion Proton Sequencer (ThermoFisher Scientific, Waltham, US), resulting in a minimum of 1 million high-quality reads of 150 bp on average per library.

#### Microbial gene count table

Filtered low-quality reads using AlienTrimmer were excluded.^69^ Alignment of reads to the human genome (accession number: GCA_009914755.4) was performed with Bowtie2, and reads with an identity exceeding 95% were removed.^70^ Subsequently, the reads were aligned to the Integrated Gut Catalogue 2 (IGC2), which encompasses 10.4 × 106 microbial genes, utilizing METEOR70.^71^ Initially, reads uniquely mapped (i.e., mapped to a single gene in the catalog) were allocated to their corresponding genes. Subsequent to this, reads that were shared (i.e., mapped to multiple genes in the catalog with the same alignment score) were assigned based on the proportion of their unique mapping counts for the genes captured. The resultant count table underwent additional processing with the utilization of the R package MetaOMineR v1.31.^71^ To address discrepancies in sequencing depth, the data was rarefied to 1 million high-quality reads. Following this, normalization for gene length was carried out on the matrix, and subsequently transformed into a frequency matrix using the fragments per kilobase of exon model per million reads mapped normalization technique.

#### Metagenomic species profiles

The IGC2 catalog was previously organized into 1990 MSPs (Metagenomic Structural Proteins), each cluster containing at least 100 genes, using MSPminer.^72^ The Genome Taxonomy Database was used to assign the taxonomy of these MSPs.^73^ The relative abundance of an MSP was determined by averaging the abundance of its 100 “marker” genes, which are the genes showing the highest collective correlation. If fewer than 10% of these “marker” genes were detected in a sample, the MSP’s abundance was set to zero. The relative abundances at higher taxonomic levels were calculated by summing the abundances of MSPs belonging to the respective taxa. The sample richness, or MSP count, was defined as the number of MSPs present in the sample, specifically those with a strictly positive abundance.

### Metabolomic analyses

#### Serum NMR sample preparation

The serum samples were thawed at room temperature before being used. Each 300 μl aliquot of serum was mixed with 300 μl of phosphate buffer at pH 7.4 (composed of 160 mm Na2HPO4, 30 mm NaH2PO4, and 3 mm NaN3 in 100% D2O).^74^ The analyses were conducted in 5 mm NMR tubes, each containing 550 µl of the prepared sample mixture. Samples were stored at 4°C until they were analyzed.

#### NMR acquisition

NMR experiments were conducted using a 700 MHz Bruker NMR spectrometer equipped with a 5 mm TCI cryoprobe at a temperature of 30.0°C. A cooled SampleJet autosampler facilitated high-throughput data acquisition. Each sample was subjected to a standard 1 h-1D NMR pulse sequence using nuclear Overhauser effect spectroscopy (NOESY) with z-gradient and water presaturation (Bruker program noesygppr1d). The experiments recorded a total of 256 transient free induction decays (FIDs) with a spectral width of 20 ppm and a relaxation delay of 4 seconds. The NOESY mixing time was set to 10 milliseconds, and the 90° pulse length was determined for each sample, averaging around 13 µs. The total acquisition time for the spectrums was 26 minutes and 54 seconds.

#### NMR data processing

For the 1 h-NMR spectra, all FIDs were processed with an exponential function for a 0.3 hz line-broadening factor before performing the Fourier transformation. The spectra were then manually phased and referenced to the glucose doublet at 5.23 ppm using TopSpin 2.2 (Bruker GmbH, Rheinstetten, Germany). TSP was excluded from the data processing procedures in this study.

#### Spectra analyses

Identification of metabolites was carried out with 1D NMR data using Chenomx NMR Suite 8.0 (Chenomx Inc., Edmonton, Canada), and this was confirmed through the examination of 2D 1 h-1 h TOCSY, 1 h-13C HSQC, and 1 h J-Resolved NMR spectra collected with standard protocols. The chemical shifts recorded were compared with reference data from the Human Metabolome Database (HMDB).^75^

### Groupe size, calculations and statistical analysis

According to a previous intervention study based on wine polyphenol supplementation, a group size of 24 or 22 subjects respectively was identified to allow to detect a clinically relevant 30%-decrease in plasma LBP/sCD14 ratio (primary criterion) or a 24% decrease in plasma LBP (*p* < 0.05 and power = 0.9).^76^ Assuming a maximum loss of 20% of participants, the sample size was 30 subjects.

The total area under the curve, for postprandial responses was calculated for endotoxemia-related and metabolic parameters using the trapezoid calculation method.^77^

Data are expressed as median and Interquartile range (Q3-Q1) on graphs and include p-values from the time*group interaction in Tables 2 to 5. Delta analyses of both interventions are calculated as differences (post – pre intervention or post – pre fructose challenge). For the analysis of metabolic endotoxemia markers, including the primary endpoint (LBP/sCD14), the results were also modeled with a global delta analysis (including one-week fructose challenge). The R package ’‘Blom” was used to transform the set of variables (except for gut microbiota) by normal score transformation (inverse normal transformation) using the Elfving, Blom, van der Waerden, Tukey and rankit methods, as well as z-score transformation (standardization) and interval scaling (normalization).

Except for the gut microbiota, a mixed model test was conducted with SAS software 9.4 (SAS® Institute, Cary, 133 NC, USA) to assess the effect of the dietary intervention using two models. A linear mixed model for repeated measures, with a compound symmetry structure as the covariance structure, was used to determine whether the difference between the changes induced by 8-week MF intervention compared to Control were statistically significant. Time, period and sequence were included as fixed variables. To account for inter-subject variability and adjust for nonspecific differences, subjects were included as random effects. Moreover, another linear mixed model (integrating two time points: before and after the one-week fructose challenge) was used to assess the impact of the MF versus Control intervention on the response to the one-week fructose challenge.

Regarding gut microbiota, impact of the product on the metagenomics features (richness, MSP) was assessed using the F1-LD-F2 design in the package nparLD v2.1 for non-parametric longitudinal values.^78^

Correlations between variables were performed using Spearman’s correlations, The correlation network plot and the scatter plot were created in RStudio (R version 3.6.3, RStudio Team, Boston, MA, USA) using the R packages “ggplot2” (version 3.3.2), “tidyverse” (version 1.3.0), and “corrr” (version 0.4). The graphs were made using GraphPad Prism version 9.2.0. The set of p-values represents the time*group interaction that constitutes our evaluation criterion.

All secondary analyses not concerning the primary endpoint were exploratory, therefore unadjusted p-values are presented as described by Li et al.^79^

## Supplementary Material

Supplemental Material

## Data Availability

The raw sequencing data have been submitted to the European Nucleotide Archive (ENA) of EBI, accessible via (https://www.ebi.ac.uk/ena/browser/view/PRJEB76794) under the project ID PRJEB76794. The study protocol and the datasets created and/or examined during this study, which include anonymized participant data, will be available on reasonable request.

## Abbreviations

CM: Cardiometabolic
GM: Gut Microbiota
LPS: Lipopolysaccharide
LBP: Lipopolysaccharide-Binding Protein
sCD14: Soluble Cluster of Differentiation 14
MF: Multifunctional
CRP: C – reactive protein
sVCAM1: Soluble Vascular Cell Adhesion Molecule 1
MCP-1: Monocyte Chemoattractant Protein 1
BCAAs: Branched-Chain Amino Acids
tAUC: Total Area Under the Curve
IR: Insulin Resistance
Adipo-IR: Adipose Tissue Insulin Resistance
1 h-NMR: Proton Nuclear Magnetic Resonance Spectroscopy
SDS: Slowly Digestible Starch
PUFAs: Polyunsaturated Fatty Acids
MSP: Metagenomic Species

## References

[1] Bulló M, Casas-Agustench P, Amigó-Correig P, Aranceta J, Salas-Salvadó J. Inflammation, obesity and comorbidities: the role of diet. Public Health Nutr. 2007;10(10A):1164–22. doi:10.1017/S1368980007000663. 17903326

[2] van den Brink W, van Bilsen J, Salic K, Hoevenaars FPM, Verschuren L, Kleemann R, Bouwman J, Ronnett GV, van Ommen B, Wopereis S. Current and future nutritional strategies to modulate inflammatory dynamics in metabolic disorders. Front Nutr. 2019 [Accessed 2023 Feb 20]. 6. https://www.frontiersin.org/articles/10.3389/fnut.2019.00129. 10.3389/fnut.2019.00129 PMC6718105 31508422

[3] Cardiovascular disease (CVD) | world heart federation. [Accessed 2024 Jan 23]. https://world-heart-federation.org/what-is-cvd/.

[4] Cani PD, Van Hul M. Gut microbiota in overweight and obesity: crosstalk with adipose tissue. Nat Rev Gastroenterol Hepatol. 2024;21(3):164–183. doi:10.1038/s41575-023-00867-z. 38066102

[5] Domingo E, Marques P, Francisco V, Piqueras L, Sanz MJ. Targeting systemic inflammation in metabolic disorders. A therapeutic candidate for the prevention of cardiovascular diseases? Pharmacol Res. 2024 Jan 11. 200:107058. Published online. doi:10.1016/j.phrs.2024.107058. 38218355

[6] Le Chatelier E, Nielsen T, Qin J, Prifti E, Hildebrand F, Falony G, Almeida M, Arumugam M, Batto J-M, Kennedy S, et al. Richness of human gut microbiome correlates with metabolic markers. Nature. 2013;500(7464):541–546. doi:10.1038/nature12506. 23985870

[7] Cani PD, Amar J, Iglesias MA, Poggi M, Knauf C, Bastelica D, Neyrinck AM, Fava F, Tuohy KM, Chabo C, et al. Metabolic endotoxemia initiates obesity and insulin resistance. Diabetes. 2007;56(7):1761–1772. doi:10.2337/db06-1491. 17456850

[8] Minihane AM, Vinoy S, Russell WR, Baka A, Roche HM, Tuohy KM, Teeling JL, Blaak EE, Fenech M, Vauzour D, et al. Low-grade inflammation, diet composition and health: current research evidence and its translation. Br J Nutr. 2015;114(7):999–1012. doi:10.1017/S0007114515002093. 26228057 PMC4579563

[9] Wunderle C, Stumpf F, Schuetz P. Inflammation and response to nutrition interventions. JPEN J Parenter Enter Nutr. 2024;48(1):27–36. doi:10.1002/jpen.2534. 38193635

[10] Hornero-Ramirez H, Aubin A, Michalski MC, Vinoy S, Caussy C, Nazare JA. Multifunctional dietary interventions, low-grade inflammation and cardiometabolic profile: a scoping review. Front Immunol. 2024 [Accessed 2024 Mar 1]. 15. doi:10.3389/fimmu.2024.1304686. PMC10927766 38476230

[11] Vors C, Nazare JA, Michalski MC, Laville M. Intérêt de la phase postprandiale pour la santé de l’Homme. Obésité. 2014;9(1):31–41. doi:10.1007/s11690-013-0410-9. 24672980

[12] Hornero-Ramirez H, Vors C, Nazare JA, Michalski MC. L’inflammation postprandiale. Prat En Nutr. 2024;20(77):8–11. doi:10.1016/j.pranut.2023.12.003.

[13] Lambert-Porcheron S, Normand S, Blond E, Sothier M, Roth H, Meynier A, Vinoy S, Laville M, Nazare J-A. Modulation of starch digestibility in breakfast cereals consumed by subjects with metabolic risk: impact on markers of oxidative stress and inflammation during fasting and the postprandial period. Mol Nutr Food Res. 2017;61(12). doi:10.1002/mnfr.201700212. 28853210

[14] Vors C, Pineau G, Drai J, Meugnier E, Pesenti S, Laville M, Laugerette F, Malpuech-Brugère C, Vidal H, Michalski M-C. Postprandial endotoxemia linked with chylomicrons and lipopolysaccharides handling in obese versus lean men: a lipid dose-effect trial. J Clin Endocrinol Metab. 2015;100(9):3427–3435. doi:10.1210/JC.2015-2518. 26151336

[15] Anhê FF, Nachbar RT, Varin TV, Vilela V, Dudonné S, Pilon G, Fournier M, Lecours M-A, Desjardins Y, Roy D, et al. A polyphenol-rich cranberry extract reverses insulin resistance and hepatic steatosis independently of body weight loss. Mol Metab. 2017;6(12):1563–1573. doi:10.1016/j.molmet.2017.10.003. 29107524 PMC5699918

[16] Nazare JA, de Rougemont A, Normand S, Sauvinet V, Sothier M, Vinoy S, Désage M, Laville M. Effect of postprandial modulation of glucose availability: short- and long-term analysis. Br J Nutr. 2010;103(10):1461–1470. doi:10.1017/S0007114509993357. 20030904

[17] Paquette M, Medina Larqué AS, Weisnagel SJ, Desjardins Y, Marois J, Pilon G, Dudonné S, Marette A, Jacques H. Strawberry and cranberry polyphenols improve insulin sensitivity in insulin-resistant, non-diabetic adults: a parallel, double-blind, controlled and randomised clinical trial. Br J Nutr. 2017;117(4):519–531. doi:10.1017/S0007114517000393. 28290272 PMC5426341

[18] Amiot MJ, Riva C, Vinet A. Effects of dietary polyphenols on metabolic syndrome features in humans: a systematic review. Obes Rev Off J Int Assoc Study Obes. 2016;17(7):573–586. doi:10.1111/obr.12409. 27079631

[19] Anhê FF, Choi BSY, Dyck JRB, Schertzer JD, Marette A. Host-microbe interplay in the cardiometabolic benefits of dietary polyphenols. Trends Endocrinol Metab TEM. 2019;30(6):384–395. doi:10.1016/j.tem.2019.04.002. 31076221

[20] Meynier M, Daugey V, Mallaret G, Gervason S, Meleine M, Barbier J, Aissouni Y, Lolignier S, Bonnet M, Ardid D, et al. Pasteurized akkermansia muciniphila improves irritable bowel syndrome-like symptoms and related behavioral disorders in mice. Gut Microbes. 2024;16(1):2298026. doi:10.1080/19490976.2023.2298026. 38170633 PMC10766393

[21] Depommier C, Everard A, Druart C, Plovier H, Van Hul M, Vieira-Silva S, Falony G, Raes J, Maiter D, Delzenne NM, et al. Supplementation with akkermansia muciniphila in overweight and obese human volunteers: a proof-of-concept exploratory study. Nat Med. 2019;25(7):1096–1103. doi:10.1038/s41591-019-0495-2. 31263284 PMC6699990

[22] Everard A, Belzer C, Geurts L, Ouwerkerk JP, Druart C, Bindels LB, Guiot Y, Derrien M, Muccioli GG, Delzenne NM, et al. Cross-talk between akkermansia muciniphila and intestinal epithelium controls diet-induced obesity. Proc Natl Acad Sci USA. 2013;110(22):9066–9071. doi:10.1073/pnas.1219451110. 23671105 PMC3670398

[23] Rodríguez-Daza MC, de Vos WM. Polyphenols as drivers of a homeostatic gut microecology and immuno-metabolic traits of akkermansia muciniphila: from mouse to man. Int J Mol Sci. 2022;24(1):45. doi:10.3390/ijms24010045. 36613488 PMC9820369

[24] Vors C, Le Barz M, Bourlieu C, Michalski MC. Dietary lipids and cardiometabolic health: a new vision of structure–activity relationship. Curr Opin Clin Nutr Metab Care. 2020;23(6):451. doi:10.1097/MCO.0000000000000693. 32889824

[25] Vinoy S, Normand S, Meynier A, Sothier M, Louche-Pelissier C, Peyrat J, Maitrepierre C, Nazare J-A, Brand-Miller J, Laville M. Cereal processing influences postprandial glucose metabolism as well as the GI effect. J Am Coll Nutr. 2013;32(2):79–91. doi:10.1080/07315724.2013.789336. 24015715 PMC4673596

[26] Vinoy S, Meynier A, Goux A, Jourdan-Salloum N, Normand S, Rabasa-Lhoret R, Brack O, Nazare J-A, Péronnet F, Laville M. The effect of a breakfast rich in slowly digestible starch on glucose metabolism: a statistical meta-analysis of randomized controlled trials. Nutrients. 2017;9(4):318. doi:10.3390/nu9040318. 28333086 PMC5409657

[27] Nazare JA, Sauvinet V, Normand S, Guérin-Deremaux L, Gabert L, Désage M, Wils D, Laville M. Impact of a resistant dextrin with a prolonged oxidation pattern on day-long ghrelin profile. J Am Coll Nutr. 2011;30(1):63–72. doi:10.1080/07315724.2011.10719945. 21697540

[28] Aliasgharzadeh A, Dehghan P, Gargari BP, Asghari-Jafarabadi M. Resistant dextrin, as a prebiotic, improves insulin resistance and inflammation in women with type 2 diabetes: a randomised controlled clinical trial. Br J Nutr. 2015;113(2):321–330. doi:10.1017/S0007114514003675. 27028002

[29] Fernandes R, Do Rosario VA, Mocellin MC, Kuntz MGF, Trindade EBSM. Effects of inulin-type fructans, galacto-oligosaccharides and related synbiotics on inflammatory markers in adult patients with overweight or obesity: a systematic review. Clin Nutr Edinb Scotl. 2017;36(5):1197–1206. doi:10.1016/j.clnu.2016.10.003. 27771020

[30] Demangeat A, Hornero-Ramirez H, Meynier A, Sanoner P, Atkinson FS, Nazare J-A, Vinoy S. Complementary nutritional improvements of cereal-based products to reduce postprandial glycemic response. Nutrients. 2023;15(20):4401. doi:10.3390/nu15204401. 37892479 PMC10609865

[31] Twardowska A, Makaro A, Binienda A, Fichna J, Salaga M. Preventing bacterial translocation in patients with leaky gut syndrome: nutrition and pharmacological treatment options. Int J Mol Sci. 2022;23(6):3204. doi:10.3390/ijms23063204. 35328624 PMC8949204

[32] Akiba Y, Maruta K, Takajo T, Narimatsu K, Said H, Kato I, Kuwahara A, Kaunitz JD. Lipopolysaccharides transport during fat absorption in rodent small intestine. Am J Physiol Gastrointest Liver Physiol. 2020;318(6):G1070–G1087. doi:10.1152/ajpgi.00079.2020. 32390462 PMC7311662

[33] Bailey MA, Holscher HD. Microbiome-mediated effects of the Mediterranean diet on inflammation. Adv Nutr Bethesda Md. 2018;9(3):193–206. doi:10.1093/advances/nmy013. PMC5952955 29767701

[34] Mohammad S, Thiemermann C. Role of metabolic endotoxemia in systemic inflammation and potential interventions. Front Immunol. 2020;11:594150. doi:10.3389/fimmu.2020.594150. 33505393 PMC7829348

[35] Mani V, Hollis JH, Gabler NK. Dietary oil composition differentially modulates intestinal endotoxin transport and postprandial endotoxemia. Nutr Metab. 2013;10(1):6. doi:10.1186/1743-7075-10-6. PMC3577458 23305038

[36] Beam A, Clinger E, Hao L. Effect of diet and dietary components on the composition of the gut microbiota. Nutrients. 2021;13(8):2795. doi:10.3390/nu13082795. 34444955 PMC8398149

[37] Seethaler B, Basrai M, Neyrinck AM, Nazare J-A, Walter J, Delzenne NM, Bischoff SC. Biomarkers for assessment of intestinal permeability in clinical practice. Am J Physiol Gastrointest Liver Physiol. 2021;321(1):G11–G17. doi:10.1152/ajpgi.00113.2021. 34009040

[38] Ricciuto A, Griffiths AM. Clinical value of fecal calprotectin. Crit Rev Clin Lab Sci. 2019;56(5):307–320. doi:10.1080/10408363.2019.1619159. 31088326

[39] Mendall MA, Chan D, Patel R, Kumar D. Faecal calprotectin: factors affecting levels and its potential role as a surrogate marker for risk of development of Crohn’s disease. BMC Gastroenterol. 2016;16(1):126. doi:10.1186/s12876-016-0535-z. 27717310 PMC5054545

[40] Fraga M, Godat S, Schoepfer AM, Moradpour D, Nydegger A. [b]Calprotectine[/b] fécale : outil diagnostique dans les maladies inflammatoires chroniques de l’intestin. Revue Médicale Suisse. 2012;8(352):1669–1673. doi:10.53738/REVMED.2012.8.352.1669. 22988727

[41] Han D, Wu Y, Lu D, Pang J, Hu J, Zhang X, Wang Z, Zhang G, Wang J. Polyphenol-rich diet mediates interplay between macrophage-neutrophil and gut microbiota to alleviate intestinal inflammation. Cell Death Dis. 2023;14(10):14. doi:10.1038/s41419-023-06190-4. 37813835 PMC10562418

[42] Seethaler B, Lehnert K, Yahiaoui-Doktor M, Basrai M, Vetter W, Kiechle M, Bischoff SC. Omega-3 polyunsaturated fatty acids improve intestinal barrier integrity—albeit to a lesser degree than short-chain fatty acids: an exploratory analysis of the randomized controlled LIBRE trial. Eur J Nutr. 2023;62(7):2779–2791. doi:10.1007/s00394-023-03172-2. 37318580 PMC10468946

[43] Usuda H, Okamoto T, Wada K. Leaky Gut: effect of dietary fiber and fats on microbiome and intestinal barrier. Int J Mol Sci. 2021;22(14):7613. doi:10.3390/ijms22147613. 34299233 PMC8305009

[44] Hokayem M, Blond E, Vidal H, Lambert K, Meugnier E, Feillet-Coudray C, Coudray C, Pesenti S, Luyton C, Lambert-Porcheron S, et al. Grape polyphenols prevent fructose-induced oxidative stress and insulin resistance in first-degree relatives of type 2 diabetic patients. Diabetes Care. 2013;36(6):1454–1461. doi:10.2337/dc12-1652. 23275372 PMC3661802

[45] Medina‐Remón A, Casas R, Tressserra‐Rimbau A, Ros E, Martínez‐González MA, Fitó M, Corella D, Salas‐Salvadó J, Lamuela‐Raventos RM, Estruch R. Polyphenol intake from a Mediterranean diet decreases inflammatory biomarkers related to atherosclerosis: a substudy of the PREDIMED trial. Br J Clin Pharmacol. 2017;83(1):114–128. doi:10.1111/bcp.12986. 27100393 PMC5338147

[46] Tan H, Zhao J, Zhang H, Zhai Q, Chen W. Novel strains of Bacteroides fragilis and Bacteroides ovatus alleviate the lps-induced inflammation in mice. Appl Microbiol Biotechnol. 2019;103(5):2353–2365. doi:10.1007/s00253-019-09617-1. 30666361

[47] Gauffin Cano P, Santacruz A, Moya Á, Sanz Y. Bacteroides uniformis CECT 7771 ameliorates metabolic and immunological dysfunction in mice with high-fat-diet induced obesity. PLoS One. 2012;7(7):e41079. doi:10.1371/journal.pone.0041079. 22844426 PMC3406031

[48] Waters JL, Ley RE. The human gut bacteria christensenellaceae are widespread, heritable, and associated with health. BMC Biol. 2019;17(1):83. doi:10.1186/s12915-019-0699-4. 31660948 PMC6819567

[49] Schoeler M, Caesar R. Dietary lipids, gut microbiota and lipid metabolism. Rev Endocr Metab Disord. 2019;20(4):461–472. doi:10.1007/s11154-019-09512-0. 31707624 PMC6938793

[50] Machate DJ, Figueiredo PS, M G, Guimarães RDCA, Hiane PA, Bogo D, Pinheiro VAZ, Oliveira LCSD, Pott A. Fatty acid diets: regulation of gut microbiota composition and obesity and its related metabolic dysbiosis. Int J Mol Sci. 2020;21(11):4093. doi:10.3390/ijms21114093. 32521778 PMC7312778

[51] Ghezzal S, Postal BG, Quevrain E, Brot L, Seksik P, Leturque A, Thenet S, Carrière V. Palmitic acid damages gut epithelium integrity and initiates inflammatory cytokine production. Biochim Biophys Acta BBA - Mol Cell Biol Lipids. 2020;1865(2):158530. doi:10.1016/j.bbalip.2019.158530. 31647994

[52] Newgard CB, An J, Bain JR, Muehlbauer MJ, Stevens RD, Lien LF, Haqq AM, Shah SH, Arlotto M, Slentz CA, et al. A branched-chain amino acid-related metabolic signature that differentiates obese and lean humans and contributes to insulin resistance. Cell Metab. 2009;9(4):311–326. doi:10.1016/j.cmet.2009.02.002. 19356713 PMC3640280

[53] Tai ES, Tan MLS, Stevens RD, Low YL, Muehlbauer MJ, Goh DLM, Ilkayeva OR, Wenner BR, Bain JR, Lee JJM, et al. Insulin resistance is associated with a metabolic profile of altered protein metabolism in Chinese and Asian-Indian men. Diabetologia. 2010;53(4):757–767. doi:10.1007/s00125-009-1637-8. 20076942 PMC3753085

[54] Gojda J, Cahova M. Gut microbiota as the link between elevated BCAA serum levels and insulin resistance. Biomolecules. 2021;11(10):1414. doi:10.3390/biom11101414. 34680047 PMC8533624

[55] Bartova S, Madrid-Gambin F, Fernández L, Carayol J, Meugnier E, Segrestin B, Delage P, Vionnet N, Boizot A, Laville M, et al. Grape polyphenols decrease circulating branched chain amino acids in overfed adults. Front Nutr. 2022;9:998044. doi:10.3389/fnut.2022.998044. 36386937 PMC9643885

[56] Ruiz-Canela M, Guasch-Ferré M, Toledo E, Clish CB, Razquin C, Liang L, Wang DD, Corella D, Estruch R, Hernáez Á, et al. Plasma branched chain/aromatic amino acids, enriched Mediterranean diet and risk of type 2 diabetes: case-cohort study within the PREDIMED trial. Diabetologia. 2018;61(7):1560–1571. doi:10.1007/s00125-018-4611-5. 29663011 PMC5988977

[57] Maltais-Payette I, Allam-Ndoul B, Pérusse L, Vohl MC, Tchernof A. Circulating glutamate level as a potential biomarker for abdominal obesity and metabolic risk. Nutr Metab Cardiovasc Dis. 2019;29(12):1353–1360. doi:10.1016/j.numecd.2019.08.015. 31668457

[58] Chaouche L, Marcotte F, Maltais-Payette I, Tchernof A. Glutamate and obesity – what is the link? Curr Opin Clin Nutr Metab Care. 2024;27(1):70. doi:10.1097/MCO.0000000000000991. 37937722

[59] Sabag A, Way KL, Keating SE, Sultana RN, O’Connor HT, Baker MK, Chuter VH, George J, Johnson NA. Exercise and ectopic fat in type 2 diabetes: a systematic review and meta-analysis. Diabetes Metab. 2017;43(3):195–210. doi:10.1016/j.diabet.2016.12.006. 28162956

[60] Bales CW, Kraus WE. Caloric restriction: implications for human cardiometabolic health. J Cardiopulm Rehabil Prev. 2013;33(4):201–208. doi:10.1097/HCR.0b013e318295019e. 23748374 PMC3696577

[61] Adriouch S, Lampuré A, Nechba A, Baudry J, Assmann K, Kesse-Guyot E, Hercberg S, Scalbert A, Touvier M, Fezeu LK. Prospective association between total and specific dietary polyphenol intakes and cardiovascular disease risk in the nutrinet-santé French cohort. Nutrients. 2018;10(11):1587. doi:10.3390/nu10111587. 30380657 PMC6266343

[62] WHO updates guidelines on fats and carbohydrates. [Accessed 2024 Mar 7]. https://www.who.int/news/item/17-07-2023-who-updates-guidelines-on-fats-and-carbohydrates.

[63] Ranaivo H, Thirion F, Béra-Maillet C, Guilly S, Simon C, Sothier M, Van Den Berghe L, Feugier-Favier N, Lambert-Porcheron S, Dussous I, et al. Increasing the diversity of dietary fibers in a daily-consumed bread modifies gut microbiota and metabolic profile in subjects at cardiometabolic risk. Gut Microbes. 2022;14(1):2044722. doi:10.1080/19490976.2022.2044722. 35311446 PMC8942430

[64] Wopereis S, Stroeve JHM, Stafleu A, Bakker GCM, Burggraaf J, van Erk MJ, Pellis L, Boessen R, Kardinaal AAF, van Ommen B. Multi-parameter comparison of a standardized mixed meal tolerance test in healthy and type 2 diabetic subjects: the PhenFlex challenge. Genes Nutr. 2017;12(1):21. doi:10.1186/s12263-017-0570-6. 28861127 PMC5576306

[65] Laugerette F, Vors C, Alligier M, Pineau G, Drai J, Knibbe C, Morio B, Lambert-Porcheron S, Laville M, Vidal H, et al. Postprandial endotoxin transporters LBP and sCD14 differ in obese vs. Overweight and normal weight men during fat-rich meal digestion. Nutrients. 2020;12(6):E1820. doi:10.3390/nu12061820. PMC7353369 32570947

[66] General LPS ELISA kit | biorbyt. [Accessed 2024 Jan 30]. https://www.biorbyt.com/general-lps-elisa-kit-orb440611.html.

[67] Breyton AE, Goux A, Lambert-Porcheron S, Meynier A, Sothier M, VanDenberghe L, Brack O, Disse E, Laville M, Vinoy S, et al. Starch digestibility modulation significantly improves glycemic variability in type 2 diabetic subjects: a pilot study. Nutr Metab Cardiovasc Dis. 2021;31(1):237. doi:10.1016/j.numecd.2020.08.010. 32988721

[68] MGP SOP 001 V1. MetaGenoPolis. [Accessed 2024 Jan 24]. https://mgps.eu/sops/mgp-sop-001-v1/.

[69] Criscuolo A, Brisse S. AlienTrimmer: a tool to quickly and accurately trim off multiple short contaminant sequences from high-throughput sequencing reads. Genomics. 2013;102(5):500–506. doi:10.1016/j.ygeno.2013.07.011. 23912058

[70] Fast gapped-read alignment with bowtie 2 | nature methods. [Accessed 2024 Jan 23]. https://www.nature.com/articles/nmeth.1923. 10.1038/nmeth.1923 PMC3322381 22388286

[71] metagenopolis/momr · GitLab. GitLab. 2023 June 6 [Accessed 2024 Jan 23]. https://forgemia.inra.fr/metagenopolis/momr.

[72] Mspminer: abundance-based reconstitution of microbial pan-genomes from shotgun metagenomic data | bioinformatics | oxford academic. [Accessed 2024 Jan 23]. https://academic.oup.com/bioinformatics/article/35/9/1544/5106712. 10.1093/bioinformatics/bty830 PMC6499236 30252023

[73] GTDB - Genome taxonomy database. [Accessed 2024 Jan 23]. https://gtdb.ecogenomic.org/.

[74] Beckonert O, Keun HC, Ebbels TMD, Bundy J, Holmes E, Lindon JC, Nicholson JK. Metabolic profiling, metabolomic and metabonomic procedures for NMR spectroscopy of urine, plasma, serum and tissue extracts. Nat Protoc. 2007;2(11):2692–2703. doi:10.1038/nprot.2007.376. 18007604

[75] HMDB 4.0: the human metabolome database for 2018 | nucleic acids research | oxford academic. [Accessed 2024 Jan 29]. https://academic.oup.com/nar/article/46/D1/D608/4616873. 10.1093/nar/gkx1089 PMC5753273 29140435

[76] Clemente-Postigo M, Queipo-Ortuño MI, Boto-Ordoñez M, Coin-Aragüez L, Roca-Rodriguez MDM, Delgado-Lista J, Cardona F, Andres-Lacueva C, Tinahones FJ. Effect of acute and chronic red wine consumption on lipopolysaccharide concentrations. Am J Clin Nutr. 2013;97(5):1053–1061. doi:10.3945/ajcn.112.051128. 23576043

[77] Yeh ST. Using trapezoidal rule for the area under a curve calculation Proceedings of the 27th Annual SAS® User Group International (SUGI’02), 1-5. 2002.

[78] Noguchi K, Latif M, Thangavelu K, Konietschke F, Gel YR, Brunner E. NparLD: nonparametric analysis of longitudinal data in factorial experiments. 2022 [Accessed 2024 Jan 23]. https://cran.r-project.org/web/packages/nparLD/index.html.

[79] Li G, Taljaard M, Van den Heuvel ER, Levine MA, Cook DJ, Wells GA, Devereaux PJ, Thabane L. An introduction to multiplicity issues in clinical trials: the what, why, when and how. Int J Epidemiol. 2017;46(2):746–755. doi:10.1093/ije/dyw320. 28025257

