# Supplementary figures and images for: Multifunctional dietary approach reduces intestinal inflammation in relation with changes in gut microbiota composition in subjects at cardiometabolic risk: the SINFONI project
Source: Gut Microbes. 2024 Dec 22;17(1):2438823. doi: 10.1080/19490976.2024.2438823 (PMC12931706; doi:10.1080/19490976.2024.2438823)

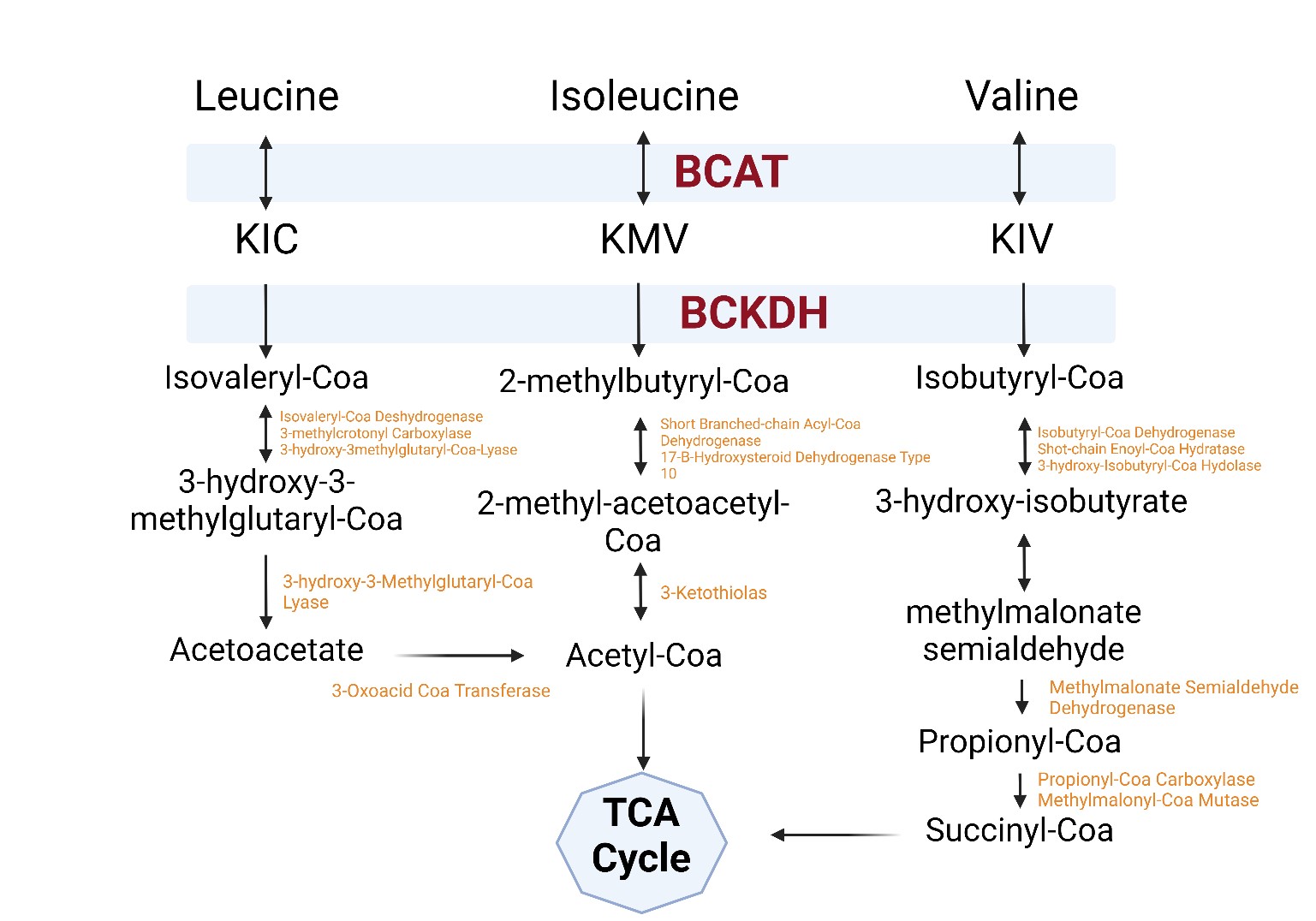

Supplement: Supplemental Material [file KGMI_A_2438823_SM7536.zip › Figure S1.jpg]

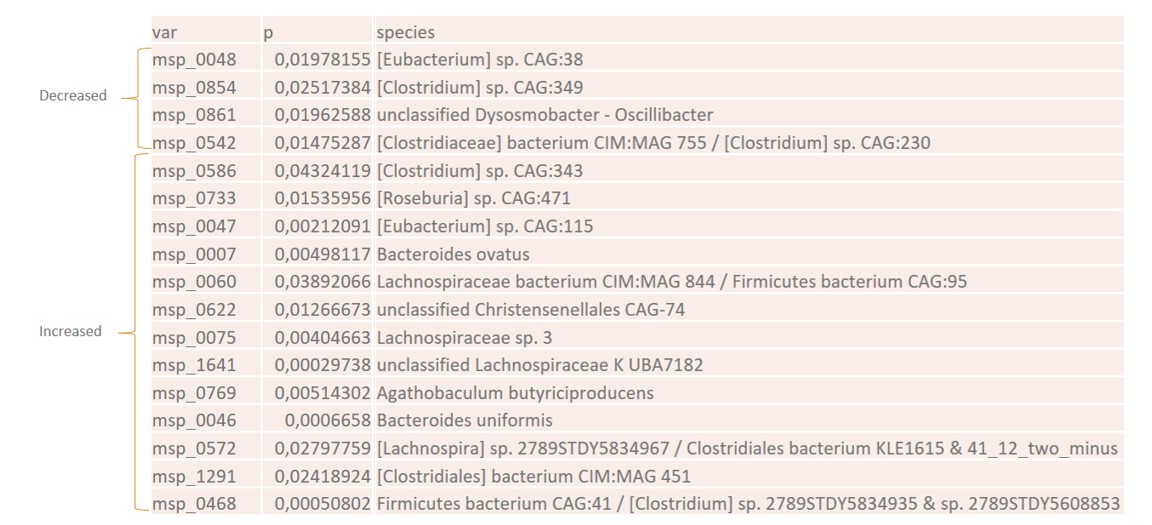

Supplement: Supplemental Material [file KGMI_A_2438823_SM7536.zip › Figure S2.jpg]

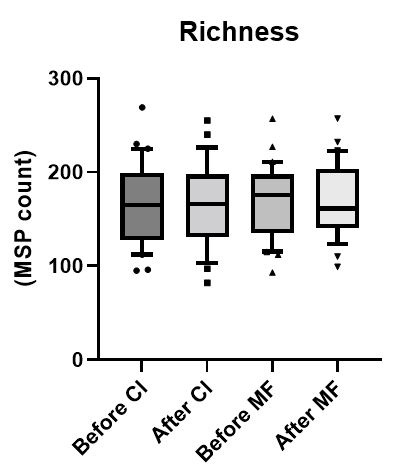

Supplement: Supplemental Material [file KGMI_A_2438823_SM7536.zip › Figure S3.JPG]
